# Evading the host response: *Staphylococcus* “hiding” in cortical bone canalicular system causes increased bacterial burden

**DOI:** 10.1038/s41413-020-00118-w

**Published:** 2020-12-10

**Authors:** Stephen D. Zoller, Vishal Hegde, Zachary D. C. Burke, Howard Y. Park, Chad R. Ishmael, Gideon W. Blumstein, William Sheppard, Christopher Hamad, Amanda H. Loftin, Daniel O. Johansen, Ryan A. Smith, Marina M. Sprague, Kellyn R. Hori, Samuel J. Clarkson, Rachel Borthwell, Scott I. Simon, Jeff F. Miller, Scott D. Nelson, Nicholas M. Bernthal

**Affiliations:** 1 Department of Orthopedic Surgery, University of California, Los Angeles, 1250 16th St Suite 2100, Santa Monica, CA 90404 USA; 2 David Geffen School of Medicine, University of California, Los Angeles, 10833 Le Conte Ave, Los Angeles, CA 90095 USA; 3 Department of Internal Medicine, University of California, Los Angeles, 757 Westwood Plaza, Suite 7501, Los Angeles, CA 90095 USA; 4 Department of Biomedical Engineering, University of California, Davis, Davis, CA 95616 USA; 5 California NanoSystems Institute, University of California, Los Angeles, 570 Westwood Plaza, Los Angeles, CA 90095 USA; 6 Department of Microbiology, Immunology, and Molecular Genetics, David Geffen School of Medicine, University of California, Los Angeles, 90095 USA; 7 Department of Pathology, University of California, Los Angeles, 1250 16th St Suite 3450, Santa Monica, CA 90404 USA

**Keywords:** Pathogenesis, Bone, Bone cancer

## Abstract

Extremity reconstruction surgery is increasingly performed rather than amputation for patients with large-segment pathologic bone loss. Debate persists as to the optimal void filler for this “limb salvage” surgery, whether metal or allograft bone. Clinicians focus on optimizing important functional gains for patients, and the risk of devastating implant infection has been thought to be similar regardless of implant material. Recent insights into infection pathophysiology are challenging this equipoise, however, with both basic science data suggesting a novel mechanism of infection of *Staphylococcus aureus* (the most common infecting agent) into the host lacunar–canaliculi network, and also clinical data revealing a higher rate of infection of allograft over metal. The current translational study was therefore developed to bridge the gap between these insights in a longitudinal murine model of infection of allograft bone and metal. Real-time *Staphylococci* infection characteristics were quantified in cortical bone vs metal, and both microarchitecture of host implant and presence of host immune response were assessed. An orders-of-magnitude higher bacterial burden was established in cortical allograft bone over both metal and cancellous bone. The establishment of immune-evading microabscesses was confirmed in both cortical allograft haversian canal and the submicron canaliculi network in an additional model of mouse femur bone infection. These study results reveal a mechanism by which *Staphylococci* evasion of host immunity is possible, contributing to elevated risks of infection in cortical bone. The presence of this local infection reservoir imparts massive clinical implications that may alter the current paradigm of osteomyelitis and bulk allograft infection treatment.

## Introduction

Over the past four decades, “limb salvage” surgery, or surgery in which bulk reconstruction is needed after a large amount of bone is removed for oncologic, infectious, or traumatic pathology, has become more commonplace than amputation. While debates have raged in the clinical communities as to whether metal^1,2^ or allograft bone^3–5^ provided a better material for reconstruction, most of the evidence provided was focused on mechanical function and durability.^6–11^ Despite a plethora of evidence demonstrating that infection is the primary cause of failure irrespective of material,^8,12^ infection has never been a driver in the debate among implants because bacterial colonization and downstream infection were always assumed to be equivalent among avascular implant materials.

Given our dependence on retrospective clinical series that vary in quality and size, a wide range of infection rates have been reported in both bulk allograft and endoprostheses (5%–30%).^3–5,13–17^ Debate remains even in a recent international consensus meeting on implant infection, with few consensus agreements across a variety of questions on periprosthetic infection diagnosis, timing, prevention, and treatment.^18^ Mechanistically, it is thought that implant materials provide an avascular surface upon which *Staphylococcus* sp., the most frequent infecting agent, can establish biofilms, enabling the evasion of immune responses and antimicrobials.^17,19,20^ These infections can be devastating, and jeopardize both limb and life of the patient.^6,7,10,13,21–23^ Based upon this data, similar infection treatment principles for both implant materials have been used for decades.^9,13,18,21^

Recent insights, however, challenge this presumed equipoise. In one of the largest series comparing allograft to metal, Albergo et al.^24^ showed a two-fold higher infection rate for allograft in a retrospective review of 133 patients from two major tumor centers treated with either bulk allograft or metal endoprostheses. Novel insights into mechanisms of Staphylococcal immune evasion in the osseous environment have complemented these clinical findings. Traditional notions of biofilm formation have focused on the formation of glycocalyx on implants, as well as the formation of staphylococcal abscess communities that shelter pathogens from the host immune response. Recently, however, the observation of Staphylococcal invasion and colonization of the lacuna-canalicular system of cortical bone have provided novel insights into the mechanisms by which these bacteria evade the host immune response in bone.^25^ Originally observed in preclinical models of osteomyelitis,^25^ the invasion of Staphylococci into submicron canalicular channels that are too small for host immune cells to penetrate has also been observed in clinical samples of chronic osteomyelitis.^26–29^ While this finding has not been widely reproduced, these data suggest that microarchitecture of bone and implant may contribute to the ability of bacteria to evade the host immune system through refuge in the submicron lacunar–canalicular system.

Finally, new evidence produced by Ghimire et al.^30^ demonstrates the importance of bioburden, as a ratio of bacteria compared to the number of available neutrophils, in overwhelming the host immune system to establish a biofilm-associated infection. Summarized simply, at key early time points of exposure, a high ratio of neutrophils to bacteria seemed to clear the implant of colonization; whereas high bacteria to neutrophil ratio overwhelmed the system and allowed a biofilm to form. Once bacteria at the low inoculum were given a “head start,” or time to establish themselves on the implant, even a high ratio of neutrophils could not eradicate the infection.

Together, these recent findings suggest that the topographical geometry of an implanted material may be of critical importance, as submicron channels provide an impassable safe haven for bacteria giving them the head start to establish a biofilm and become protected from critical early host neutrophil response. In light of this translational problem, the present study was developed in order to interrogate this hypothesis. First, we compared real-time bacterial burden on a nonporous metal implant to that on a bulked allograft implant with submicron porous channels in a murine model that permits noninvasive, longitudinal quantification of bacterial burden. Second, we added a cancellous bone implant model with macroscopic porosity and compared it to metal and cortical allograft to determine whether the microarchitecture of cortical bone is the driver of this increased bacterial colonization. Finally, we tested this hypothesis within living bone, in a separate model of mouse femur infection, to assess whether a viable blood supply would negate this proposed immune reservoir. In addition to refocusing the omnipresent clinical debate of what is the preferred implant in limb salvage surgery, furthering the mechanistic understanding of how *Staphylococcus aureus* evades the host immune system in different microenvironments has massive clinical implications on resistance formation, antibiotic/antimicrobial selection, and surgical technique for debridement. If we can implicate this lacunar–canalicular network in infection propensity, we can begin to address the largest remaining hurdle in limb salvage.

## Results

### In vivo bioluminescence of allograft implant infection

**Fig. 1:**
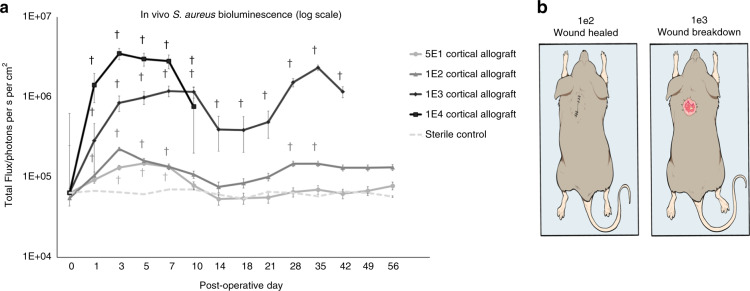
Quantitative real-time bacterial burden on cortical allograft at increasing inoculums in a mouse model. **a** Measurement of bacterial burden using in vivo bioluminescence. *S*. *aureus* possessing the bioluminescent construct in a stable plasmid (Xen36) in four inoculums [5 × 10^1^, 1 × 10^2^, 1 × 10^3^, 1 × 10^4^ colony-forming units (CFU)] or no bacteria as a control were inoculated on a cortical allograft implanted into mice. Bacterial counts as measured by in vivo *S*. *aureus* bioluminescence [total flux (photons per s per cm^2^)sem (logarithmic scale)]. Error bars represent standard error of the mean. ^†^*P* < 0.05 compared to sterile in the mixed effects regression model using a group-by-time interaction term. Experiment was carried out through postoperative day (POD) 56 in order to identify an end plateau to the main curve of interest, the 1 × 10^2^ inoculum. **b** Cortical allograft surgery. Mouse on left inoculated with 1 × 10^2^ CFUs *S*. *aureus* had a healed surgical wound on POD21. Mouse on right inoculated with 1 × 10^3^ CFUs *S*. *aureus* had wound breakdown necessitating euthanasia on POD21

Bulk allograft, stainless steel, and cancellous allograft implantation in mice were successfully performed (Supplementary Fig. 1). Bulk allograft inoculated with *S. aureus* had a concentration dependent increase in bioluminescent signal that initially peaked between postoperative day (POD) 3–10 (Fig. 1 and Supplementary Fig. 2). Infected bulk allograft mice demonstrated a second increase in bioluminescent signal, beginning on POD 18 and peaking on POD 35, suggesting a second, delayed increase in infection intensity (Fig. 1). Inoculums with 1 × 10^2^ CFUs were statistically significantly different from control at all curve peak points (*P* < 0.05). The lower inoculum, 5 × 10^1^, did not achieve statistically significant difference from sterile control after POD 7 (Fig. 1).

### Bacterial inoculum on allograft and wound breakdown

All allograft mice in the 1 × 10^4^ group and 50% of mice in the 1 × 10^3^ group developed wound breakdown and had to be euthanized (Fig. 1). In the 5 × 10^1^ group, final infection level of 7.8 × 10^4^ p/s/cm^2^/sr at POD 56 was not significantly different from control (*P* = 0.60). There was no evidence of wound breakdown in the 1 × 10^2^ group.

### In vivo bioluminescence of allograft implant infection compared to metal and cancellous allograft

Both stainless steel discs and cancellous allograft inoculated with 1 × 10^2^ or 1 × 10^3^ CFU *S. aureus* generated an initial bioluminescent peak on POD 3, suggesting initial infection, followed by resolution and maintenance of signal at control levels after POD 7 (N.S.).

**Fig. 2:**
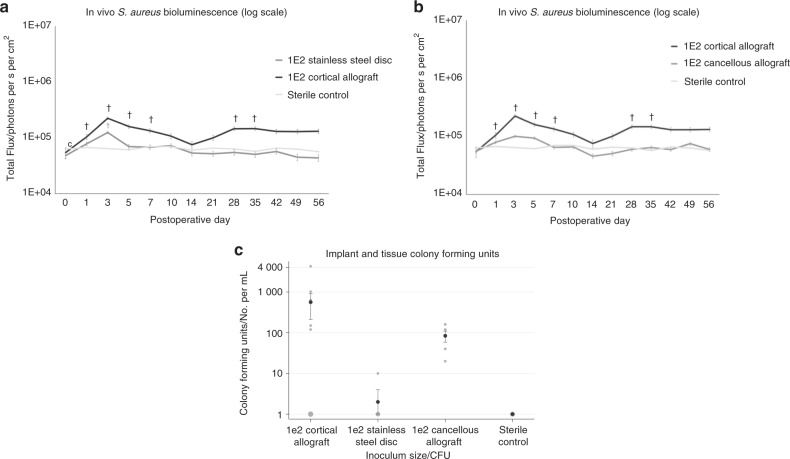
Quantitative real-time bacterial burden on cortical allograft compared to stainless steel disc or cancellous allograft in a mouse model. **a**, **b** Measurement of bacterial burden using in vivo bioluminescence. *S*. *aureus* possessing the bioluminescent construct in a stable plasmid (Xen36) in the inoculum 1 × 10^2^ CFU or no bacteria as a control were inoculated into the dorsal cervical subcutaneous space of mice in the presence of either a cortical allograft implant vs stainless steel disc (**a**), or cortical allograft implant vs cancellous allograft implant (**b**). Bacterial counts as measured by in vivo *S*. *aureus* bioluminescence [total flux (photons per s per cm^2^)sem (logarithmic scale)]. Error bars represent standard error of the mean. Full experimental data including 1 × 10^3^ CFU inoculum in Supplementary Figs. 3 and 4. ^†^*P* < 0.05 compared to sterile in the mixed effects regression model using a group-by-time interaction term. **c** Confirmation of bacterial burden using CFU counts. At POD 56 mice were sacrificed, implants were sonicated, tissue was homogenized, and bacteria was cultured and counted. Black circles represent mean. Error bars represent standard error of the mean. Gray plot points represent individual data points, and size of plot point circle is proportional to number of data points at that value. The number of bacterial CFU that were adherent to the implant and in the surrounding tissue was determined by counting CFU after overnight culture of plates, and was expressed as CFUs per mL harvested

Bulk allograft maintained a significantly higher infection intensity than stainless steel disc at the 1 × 10^2^ inoculum on first peak POD 3 (*P* < 0.000 1), second peak POD 35 (*P* = 0.012), and conclusion of experiment neared significance (*P* = 0.083) (Fig. 2a and Supplementary Fig. 3). Bulk allograft maintained a significantly higher infection intensity than cancellous allograft disc at the 1 × 10^2^ inoculum on first peak POD 3 (*P* < 0.001), second peak POD 35 (*P* = 0.010), and approached significance at the conclusion of the experiment (*P* = 0.07) (Fig. 2b and Supplementary Fig. 4). There was no evidence of wound infection in the stainless steel disc or cancellous allograft groups.

### Correlation of bioluminescence with CFU

The correlation of noninvasive imaging with CFU has previously been confirmed and validated in other models from this laboratory.^31,32^ CFU results trended with bioluminescence findings in this experiment, despite not reaching statistical significance. At the 1 × 10^2^ dose on POD 56 (Fig. 2), allograft generated higher average CFU than disc or cancellous, at 562 vs 2 (*P* = 0.13) and 562 vs 84 (*P* = 0.20). Bulk allograft CFU also demonstrated a dose response relationship, with 5 × 10^1^ vs 1 × 10^2^ inoculum generating 65 vs 562 CFU (Supplementary Fig. 5) (*P* = 0.42).

### In vivo radiographic evaluation

Radiographs taken on POD 0 confirmed proper subcutaneous placement of allograft implants and stainless steel disc (Fig. 1). Radiographs on POD 18, 35, and 56 confirmed continued presence of implants in proper position with no evidence of bone resorption (Supplementary Fig. 6).

### Ex vivo macroscopic evaluation

Macroscopic visualization of infected and sterile bulk allograft on POD 4 revealed expected postsurgical inflammatory hyperemia. Infected bulk allograft on POD 18, 35, and 56 revealed extensive vascular proliferation, erythema, and a robust capsule layer surrounding the allograft (Supplementary Fig. 7). Sterile allograft on POD 18, 35, and 56 did not demonstrate vascular proliferation, surrounding erythema, or capsule (Supplementary Fig. 7). Both cancellous allograft and stainless steel disc infected and sterile specimens revealed no differences in inflammation, vascularity, or capsular formation at any time point, with the exception of modest vascularity present at POD 18 in cancellous allograft (Supplementary Fig. 7).

### Ex vivo histologic evaluation

**Fig. 3:**
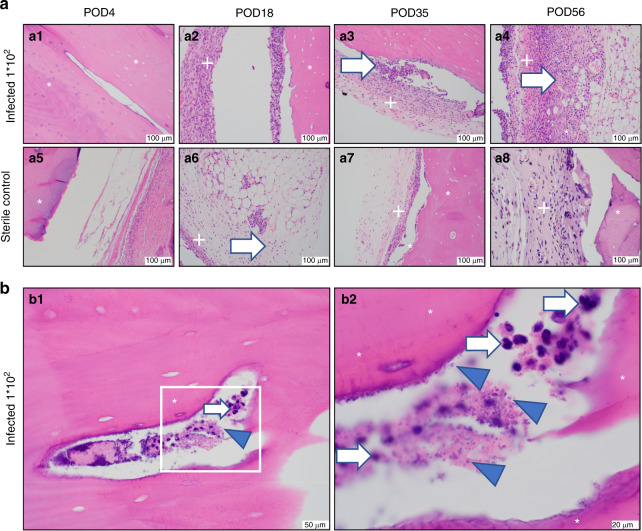
Cortical allograft histology. **a** All images taken at ×200. Hematoxylin and eosin stain. **a1–a4** Inoculated with 1 × 10^2^ CFU *S. aureus*. **a5–a8** Sterile control. **a1** POD 4, acute inflammatory cells, no capsule; **a2** POD 18, thick inflammatory capsule and evidence of neovascularization in cortical bone; **a3** POD 35, thick inflammatory capsule and evidence of neovascularization in cortical bone; **a4** POD 56, neovascular formation within thick capsule and extensive inflammatory reaction, **a5** sterile POD 4, acute inflammatory cells, no capsule; **a6** sterile POD 18; **a7** sterile POD 35, thin inflammatory capsule, no evidence of bacteria, and a multinucleated giant cell with resorption pit; **a8** sterile POD 56, thin inflammatory capsule, no evidence of bacteria. Note in particular the increased cellularity and inflammatory presence in the infected specimens compared to the sterile specimens. * = bone, + = capsule, white arrow = inflammatory cells, blue arrowhead = cocci bacteria. **b** Hematoxylin and eosin stain. Inoculated with 1 × 10^2^ CFU *S. aureus*. **b1** ×400, bacterial cocci within haversian canal. **b2** POD 56, ×1 000, inset. Bacterial cocci and inflammatory cells within haversian canal. Note in particular the presence of both cocci bacteria and inflammatory cells co-existing in the same haversian canal space. * = bone. White arrow = inflammatory cells. Blue arrowhead = cocci bacteria

**Fig. 4:**
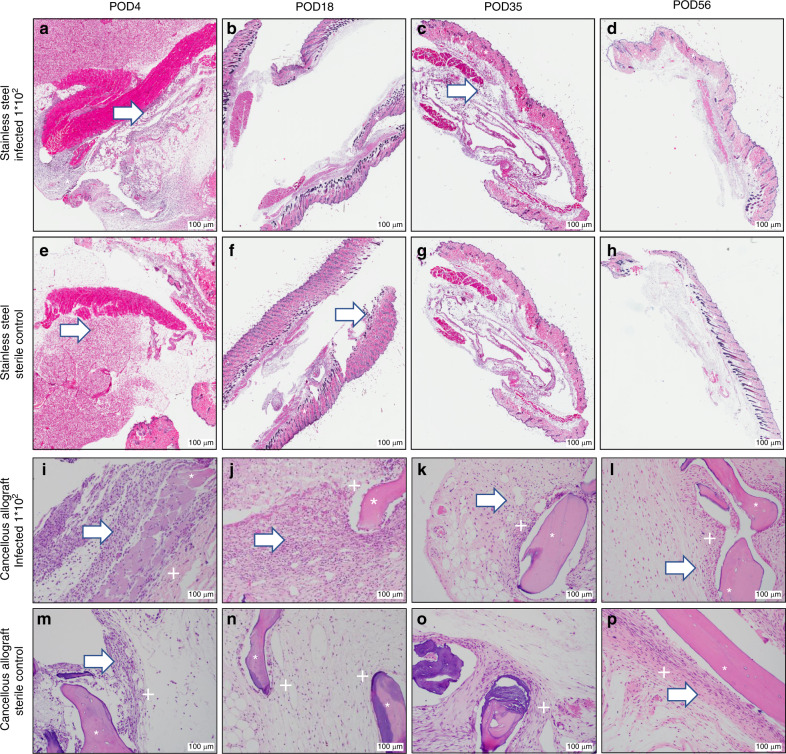
Stainless steel disc and cancellous allograft histology. All images taken at ×200. **a**–**d** Inoculated with 1 × 10^2^ CFU *S. aureus*. **e**–**h** Sterile control. **a** Acute inflammatory cells. **b** Acute inflammatory cells, no capsule. **c** No capsule, stroma tissue. **d** No capsule, stroma tissue. **e** Acute inflammatory cells. **f** Acute inflammatory cells, no capsule. **g** No capsule, stroma tissue. **h** No capsule, stroma tissue. Note in particular the presence of significant inflammatory cells throughout both infected and sterile specimens, but no evidence of cocci bacteria was discovered throughout. * = stromal tissue, + = capsule, white arrow = inflammatory cells. Cancellous allograft histology. **i**–**l** Inoculated with 1 × 10^2^ CFU *S. aureus*. **m**–**p** sterile control. **i** Thin capsule surrounding cancellous bone, neutrophils. **j** Thin capsule surrounding cancellous bone, neutrophils. **k** Thin capsule surrounding cancellous bone, neutrophils. **l** Thin capsule surrounding cancellous bone, neutrophils and eosinophils. **m** Thin capsule surrounding cancellous bone, minimal acute inflammatory cells. **n** Thin capsule surrounding cancellous bone, no acute inflammatory cells. **o** Thin capsule surrounding cancellous bone. **p** Thin capsule surrounding cancellous bone, some eosinophils. Note in particular the presence of inflammatory cells throughout both infected and sterile specimens, but no evidence of cocci bacteria was discovered throughout. * = bone, + = capsule, white arrow = inflammatory cells, blue arrowhead = cocci bacteria

Histologic examination of infected allograft specimens revealed early acute inflammatory cells, late capsular formation, and robust extra-capsular inflammatory cellular response. Gram-positive cocci bacteria were visualized within the haversian canal system at POD 56 in one specimen. Sterile specimens revealed early acute inflammatory cells, thinner capsule formation, and no cocci bacteria within haversian canal system (Fig. 3 and Supplementary Figs. 8 and 9). Infected bulk allograft specimens demonstrated comparatively higher presence of CD31 endothelial stain at all time points than sterile specimens (Supplementary Fig. 10), representing significant vascular invasion and consistent with macroscopic observation. Infected metal disc specimens revealed no capsular formation, vascular formation, or bacterial species at any time point in infected or sterile specimens (Fig. 4). Cancellous allograft specimens revealed increased inflammatory cells in infected specimens compared to sterile, but there was no evidence of bacteria (Fig. 4 and Supplementary Fig. 11).

### Live/dead confocal microscopy

**Fig. 5:**
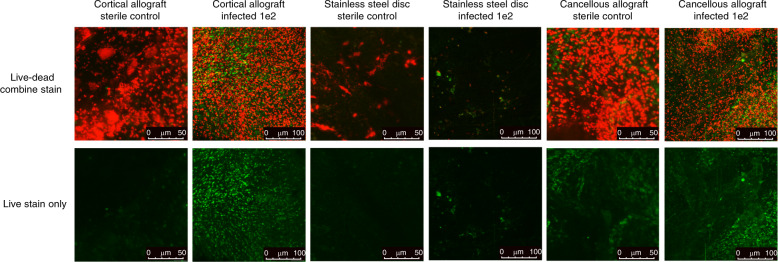
Live–dead confocal microscopy. Confocal fluorescent microscopy of cortical allograft, stainless steel disc, or cancellous allograft at POD 56 following inoculation with sterile saline, or 1 × 10^2^ CFU of *S. aureus* Xen36, magnification ×100. The commercially available Live/Dead BacLight Bacterial Viability Kit (Thermo Fischer Scientific, Canoga Park, CA) was utilized. SYTO9 dye stains nucleic acids in intact cell membranes, representing live bacteria. Propidium iodide die stains nucleic acids in ruptured cell membranes, representing dead bacteria. The comparison of the live stain in the cortical allograft reveals qualitatively more green stain/100 μm in the infected compared to the sterile group, whereas the live stains in the stainless steel and cancellous allograft appear more similar between infected and sterile groups. While the intent of the SYT09 dye is to stain microbial cells, host cells may represent a confounding factor and cautions the interpretation of this data

Fluorescent confocal microscopy revealed extensive green-fluorescent (live) and red-fluorescent (dead) bacteria on the infected cortical allograft specimens at 1 × 10^2^ inoculum. Live stain only images demonstrate the presence of live bacteria with background red-fluorescence subtracted. Sterile specimens, and infected 1 × 10^2^ stainless steel discs, revealed few live bacteria and some dead bacteria (Fig. 5). Infected and sterile cancellous allograft specimens revealed extensive green and red-fluorescent staining in both specimens (Fig. 5).

### In vivo measurement of host neutrophil response

LysEGFP mice were used to assess initial host neutrophil response to surgery. LysEGFP mice inoculated with *S. aureus* (*n* = 5) or sterile saline (*n* = 3) generated similar fluorescent peaks on POD 1–3 and had similar decay in fluorescence by POD10 (Supplementary Fig. 12a). The fluorescence peak of both sterile and infected LysEGFP mice on POD 3 was significantly different from the sterile Black6 mice (*P* = 0.006, *P* < 0.001). Enumeration of CFUs at the conclusion of the experiment confirmed infection in infected mice only (Supplementary Fig. 12b).

### In vivo native femur infection

**Fig. 6:**
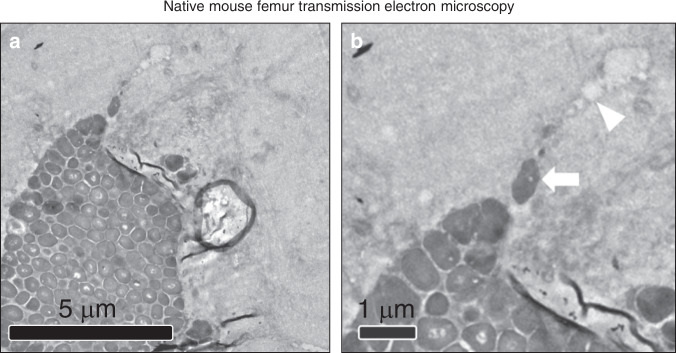
Transmission electron microscopy in native mouse femur. **a** Low power (×8 000) TEM images of Xen36 *S. aureus* invasion of the Haversian canal and adjacent canaliculus of cortical bone. **b** Enhancement of region of interest shows canaliculus (white arrowhead) with elongated *S. aureus* (white arrow) at confluence of canaliculus and Haversian canal. The discovery of elongated *S. aureus* at the confluence of the haversian canal and canaliculi supports the hypothesis that this process occurs in cortical bone in both allograft and native bone, representing a potential mechanism by which the cocci bacteria evade the host immune system

Following demonstration of *S. aureus* in cortical allograft haversian canal, experiments were repeated with host femur inoculation in a separate model (see “Materials and methods”). Mice were subjected to intramedullary inoculation with *S. aureus* (5 × 10^6^) and femurs were harvested on POD 14. TEM image in one mouse demonstrated elongated *S. aureus* located at confluence of canaliculus and Haversian canal (Fig. 6).

## Discussion

This novel translational study demonstrated increased bacterial infection burden on bulk allograft over both stainless steel and cancellous bone. All three materials are common implant choices used for reconstruction of large-segment bone defects, a clinical pathway that has largely supplanted amputation in recent decades. Unfortunately, high rates of infection persist in at-risk patients on these avascular implants. Bulk allograft infection occurs in up to 30% of cases,^3–5,13–16,33–38^ is frequently caused by *Staphylococcal* sp., and typically occurs in the 1st year after surgery.^5,13,20^ Similarly, metallic implants have traditionally been thought of as equally susceptible to infection, due to similarity in mechanistic understanding of *Staphylococcal* biofilms as well as previously published data on empiric infection rates in tumor and trauma patients.^19,39–41^ Unfortunately, however, no translational animal models currently exist to address limitations in our understanding of bulk allograft infection compared to the alternative materials.

Given this clinical dilemma, a real-time mouse model of bulk allograft infection with *S. aureus* was successfully created and generated significant findings. First, bulk allograft generated significant and chronic infections with low level inoculums of *S. aureus*. Based on this discovery, we then sought to investigate whether anatomical or material considerations in the bone affected infection characteristics, which led to comparative studies between bulk allograft and both metal and cancellous allograft. Given the unique susceptibility of only bulk allograft, we finally sought to demonstrate whether the infection characteristics persisted between cadaveric and living bone, which led to the expansion of the model into native mouse femurs.

Our finding that bulk allograft was significantly more susceptible to infection than stainless steel challenges traditional clinical thought. Bulk allograft infected with *S. aureus* generated an unexpected biphasic pattern in bacterial burden, with a late peak occurring at postoperative week 3. Neither stainless steel nor cancellous allograft experiments replicated this finding. Finally, we discovered invasion of the cortical haversian canal system by *S. aureus* only in bulk allograft, which was then replicated in the native mouse femur experiment. This microscopic phenomenon has not been observed previously in cortical bone allograft.

In contradiction with the majority of clinical data, this study demonstrated an orders-of-magnitude higher bacterial burden established on bulk allograft compared to stainless steel. Whereas inoculums as low as 1 × 10^2^ CFUs of *S. aureus* were able to establish a chronic infection on cortical bone allograft, a 10x dose of 1 × 10^3^ CFUs was cleared by the host on a stainless steel implant and cancellous bone (Fig. 2).

Supportive of this finding is the implant–inoculum interplay concept of implant infection developed by Ghimire et al.^30^ Their in vitro work demonstrated that both time before exposure to neutrophils and quantity of initial bacteria inoculation were critical determinants of successful *Staphylococci* implant infection. Novel hypotheses in neutrophil identification and phagocytosis of bacteria were explored, revealing that variation in the host–implant interface impacts the delicate balance between either bacteria persistence or eradication. Extrapolated to our work, this concept of time to exposure and quantity of neutrophil vs bacteria inoculum helps illuminate why cortical bone, with its immune-evading canaliculi network, helps cultivate a higher bacterial burden than either stainless steel or cancellous bone, which lack a submicron reservoir and are both immediately accessible to host immune response. Additionally, our in vivo neutrophil analyses revealed a similar immediate neutrophil response to initial surgical insult irrespective of bacterial burden, lending support for the theory that initial bacterial inoculum is a driver of the success rate of persistent bacterial infection (Supplementary Fig. 12). Finally, recent evidence from a multi-center retrospective study by Albergo et al.^24^ demonstrated a two-times higher infection rate in bulk allograft compared to metal endoprostheses, suggesting this increased bacterial burden established on bulk allograft compared to metal may also be borne out clinically.

The second unexpected finding in this model was the routinely replicated second peak in bacterial burden at postoperative week 3. This peak occurred in several inoculum levels, from 5 × 10^1^ to 1 × 10^3^ CFUs. This is the first description of a second, late increase in infection in bulk allograft, and ex vivo analyses corroborated in vivo imaging data. Whether this phenomenon is a result of host or infection factors remains under further investigation, and several hypotheses exist. The second peak may represent a delayed release of bacteria from the cortical bone microarchitecture corresponding to the decrease in host immune response following initial surgical insult. Alternatively, delayed development of microabscesses present late within the haversian canal and canalicular system may also be responsible for the delayed rebound in bacterial burden. Previous murine models of spine and joint implant infections have not demonstrated this second infection peak, and thus future investigation is warranted.^31,42^

This biphasic bacterial occurrence has important translational implications. Common chemotherapy protocols for treatment of osteosarcoma (a pathology commonly responsible for indication for limb salvage surgery) often prescribe immunosuppressant chemotherapy agents on 2–3-week cycles,^43,44^ potentially coinciding with the second peak in infection burden and thereby increasing the risk of infection in certain patients. These data therefore indicate that a heightened risk of delayed infection may be considered when discussing chemotherapy regimens with high-risk patients undergoing tumor resection and reconstruction with bulk allograft.

The third and final important phenomenon revealed in this study was the observation of *S. aureus* within both allograft cortical haversian canals at POD 56 (Fig. 3a, b) and native mouse femur submicron canaliculi (Fig. 6a, b). Microabscesses were observed within allograft cortical bone only at late time points (POD 56), and not early (POD 4, 18, and 35). The cortical residence of bacteria may be related to the presence of immune cell upregulation and neutrophil presence seen at POD 35 (Fig. 3a), a well-documented phenomenon in the setting of bone infection.^45^ *S. aureus* has been understood to be a nonmotile species, with capability to form biofilm, bind endothelial cells, or trigger phagocytosis by immune cells,^46–48^ but no mechanism has previously existed to explain invasion of allograft haversian system as observed here, until recently. The additional host femur inoculation model was performed in order to assess whether *S. aureus* may evade the host immune system in the face of a viable vascular supply; the discovery of elongated *S. aureus* at the confluence of the haversian canal and canaliculi supports this hypothesis (Fig. 6a, b).

A rigorous description of a proposed *S. aureus* mechanism of invasion of cortical bone was revealed by de Mesy Bentley et al.^25^, who observed, through transmission electron microscopy, *S. aureus* undergoing asymmetric binary fission in vicinity of cortical canaliculi. As their study was performed in native mouse femurs, we replicated their findings in our mouse model, revealing elongated *S. aureus* at the interface of the haversian canal and canaliculus (Fig. 6). These results both confirmed the ability of *S. aureus* to invade host canaliculi network and underscore the importance of the microarchitecture of the host implant in permitting persistent *Staphylococci* infection. While immune cells may physically engage the larger haversian system (~50 µm), the 100× smaller size of the canaliculus (~0.13–0.39 µm) may provide a physical barrier to host immune cells and therefore provides a likely mechanistic pathway by which *S. aureus* (0.5–1.0 µm) achieve a “hiding” place from the host immune system, and ultimately, increased infection rates.^49–51^ Meanwhile, the pore size of the comparison implant materials studied in this experiment, medical grade 316L stainless steel (5–10 µm)^52^ and “normal” cancellous bone (300–600 µm)^53^ is perhaps permissibly large, enabling inflammatory cell infiltration and clearance of infection. Furthering this concept, Schwarz et al.^54^ describe the limits to bioavailability of antimicrobial agents within the osteocytic-canalicular network, providing additional support for a mechanism by which otherwise susceptible *S. aureus* develop resistance to host immunity and immunotherapies within the submicron canalicular network. Finally, Yang et al.^55^ recently proposed intracellular infection of osteocytes as a source of latent infection, contributing further to *S. aureus’* possible ability to “hide.”

These suggested mechanisms for evasion of the host immune response and antimicrobial treatment help explain the clinical hallmark of *S. aureus* osteomyelitis: periods of latent infection despite aggressive treatment, followed by recurrence. In light of these developments, our translational work further suggests that the microarchitecture of the inoculated implant may be a critical determining factor in the successful establishment of chronic infection. Submicron channels specific to cortical bone, which are smaller than immune cells but larger than *Staphylococci*, may be just the right size to foster *S. aureus* infection, compared to the too small, nonporous metal surface or the too-accessible macroscopically porous cancellous allograft. This geometric finding may be an important step toward highlighting cortical bone as a target for further research in osteomyelitis diagnosis and therapeutic modalities.

There are several strengths to this translational model, permitting the study of novel antimicrobial prevention strategies for bulk allograft infection. Clinically, many allograft infections are polymicrobial, including Staphylococci, Streptococci, and gram-negative bacteria.^19^ Risk factors for infection abound.^19,56^ Several in vitro models of antimicrobial allograft coatings exist, including covalently linked vancomycin,^57^ a photoactivated porphyrin coating,^58^ and antibiotic-impregnated polymer coatings.^59^ This novel animal model can therefore serve as a valuable tool to assess these modifications in vivo, permitting rapid assessment of translational effectiveness. Furthermore, the model can be used to assess the host response to an array of potential infective pathogens, and numerous bioluminescent strains exist.

There are several limitations to this model. Bulk allograft implantation surgery is complex, impacted by a variety of host and surgical factors including bone–bone healing, hardware fixation, and immunosuppressants, and this model is a vast simplification of the process. The model was streamlined to limit confounding factors. The dorsal cervical spine was chosen for implant location due to ease of access and avoidance of need for metal fixation. Therefore, simplifications were employed in order to specifically target host–implant interactions in bulk allograft infection. Additionally, the allograft chosen was a human frozen fibula allograft, making it a xenograft transplant. Frozen human allograft is clinically relevant and easily available (from otherwise discarded surgical specimens). Clinical research has shown no difference between frozen and fresh allograft implants with regards to immunogenicity or incorporation.^60^ Additionally, a weakness of the live/dead stain may be unknown specificity for microbial cells vs living cells. Finally, a criticism of the model could be the possibility of the suture acting as a nidus for infection. However, the impact of the suture appears to be negligible. A related mouse model of spinal implant infection has previously shown the location of bioluminescence off the midline rather than horizontally oriented with suture lines,^42^ and the methodology for the allograft model is to place the implant deep within a subcutaneous pocket far from the incision. The bioluminescent images routinely showed the peak infection to be located directly over the allograft, rather than over midline incision.

In conclusion, consensus is lacking on the infective susceptibility of bulk allograft vs metal in limb salvage surgery, due to historical equipoise in clinical data and an absence of animal modeling data. Recent basic science and clinical data have challenged this equipoise, and therefore a novel translational murine model of bulk allograft infection was developed. Capable of generating real-time, quantifiable data on bacterial burden, the model demonstrated orders-of-magnitude higher bacterial burden in bulk allograft infection than stainless steel. Furthermore, the model unexpectedly revealed a delayed second peak of bacterial burden at 3 weeks postop, with important and immediate implications for antimicrobial strategies and chemotherapy regimens. Finally, the novel observation of *S. aureus* residence within allograft haversian canals and canaliculi was confirmed in both living and dead cortical bone, in parallel with recent descriptions of a proposed mechanism for canalicular invasion, suggesting that the microarchitecture of the inoculated implant plays a critical role in the ongoing battle between host immune system and infecting agent. The establishment of this model will help propel future research and development of novel strategies to help combat bulk allograft infection and osteomyelitis.

## Materials and methods

### Ethics statement

All animals were handled in strict accordance with good animal practice as defined in the federal regulations set forth in the Animal Welfare Act, the 1996 Guide for the Care and Use of Laboratory Animals, PHS Policy for the Humane Care and Use of Laboratory Animals, as well as UCLA’s policies, and procedures as set forth in the UCLA Animal Care and Use Training Manual. All animal work was approved by the UCLA Chancellor’s Animal Research Committee (ARC# 2012–104–03J).

### *Staphylococcus aureus* bioluminescent strain

All experiments in this methodology were carried out in accordance with previously published protocols.^31,42,61^ *S. aureus* Xen36 (PerkinElmer, Hopkinton, MA) bacteria inoculations were used.^31,42,61^ Strain ATCC 49525 (Wright, Manassas, VA) was derived as a clinical isolate from a bacteremic patient. Metabolically active Xen36 produce a blue-green light with emission wavelength of 490 nm, due to a gram-positive optimized lux-ABCDE operon stably integrated into a large native plasmid.^61^ Previous experiments have confirmed the consistency of the Xen36 bioluminescent signal in direct correlation with bacterial burden.^32^

### Preparation of *S. aureus* for inoculation

*S. aureus* was prepared for inoculation as previously published.^31,42,61^ Of note, Xen36 can be isolated from contaminants due to possession of a kanamycin resistance selection marker on its lux operon. Therefore, 200 mg·mL^−1^ kanamycin (Sigma–Aldrich, St. Louis, MO) was added to all cultures to ensure sample purity. Xen36 was streaked onto tryptic soy agar plates (tryptic soy broth (TSB) plus 1.5% bacto agar, BD Biosciences, San Jose, CA) and cultured at 37 °C overnight. Next, single colonies of *S. aureus* were individually grown in TSB and again cultured overnight at 37 °C in a shaking incubator (240 r·min^−1^) (MaxQ 4450, ThermoFisher, Grand Island, NY). After a 2-h subculture of a 1:50 dilution of the resultant culture, mid-logarithmic phase bacteria were attained. Finally, using a centrifuge, bacterial cells were pelleted, re-suspended, and washed in phosphate-buffered saline (PBS). Bacterial inoculums (5 × 10^1^, 1 × 10^2^, 1 × 10^3^, and 1 × 10^4^ CFUs in 2 μL PBS) were approximated by measuring the absorbance at 600 nm [A600, Biomate 3 (Thermo)].

### Mice

Twelve-week-old male C57BL/six wild-type mice (Jackson Laboratories, Bar Harbor, ME) were used for all experiments. In latter experiments, 18-week-old male LysEGFP mice (S. I. Simon Laboratory, University of California, Davis, Sacramento, CA), which possess a knock-in gene for enhancement of green fluorescent protein into the lysozyme M gene within host neutrophils, were used. Utilization of these mice permits co-localization of the host neutrophil response, a surrogate for host immune response, to bacterial burden.^62,63^ Cages housed four mice at a time and mice were stored with a 12-h light and dark cycle. Standard pellet diet and water were available at all times. Veterinary staff tracked and assessed mice daily to ensure well-being of the animals during the experiment.

### Mouse surgical procedures

Human fibular cortical allograft and cancellous allograft chips were obtained from, and sterilized in the standard manner, by Muscoloskeletal Transplant Foundation (Edison, NJ).^64^ Bulk allograft was shaped into 2.5 mm diameter discs using a high-speed saw and wire cutter. Cancellous allograft chips were trimmed with scissors to 2.5 mm diameter. Scissors were used to trim rough edges, and implants were sterilized in an autoclave. Stainless steel discs of 2.5 mm diameter were selected. Survival surgery was performed in which the allograft or steel disc was implanted in the subcutaneous space dorsal to the caudal cervical spine (Supplementary Fig. 1). Mice were anesthetized via inhalation isoflurane (2%). The level of the implantation was approximated by palpating the maximal point of lordosis with minimum skin tension. A 1 cm midline incision was then made and carried down to fascia, exposing subcutaneous muscle. The dissection was directed bilaterally superficial to the paraspinal musculature, developing a subcutaneous pocket for the allograft implant. Fine-toothed forceps were used to gently place the sterile implant into the pocket created by dissection. An inoculation of 5 × 10^1^, 1 × 10^2^, 1 × 10^3^, or 1 × 10^4^ CFUs of bioluminescent Xen36 *S. aureus* in 2 μL phosphate-buffered solution or 2 μL sterile saline (control group) was pipetted onto the allograft. A single 4.0 Vicryl suture was then placed in a running fashion to approximate the skin. Quick-release buprenorphine (0.3 mg·kg^−1^) (Zoo-Pharm, Fort Collins, CO) was administered subcutaneously every 12 h for 72 h as postoperative analgesic. Placement of the implant was confirmed with high-resolution x-rays on POD 0, 18, 35, and 56 (Faxitron LX-60 DC-12 imaging system, Faxitron, Tucson, AZ). All surgeries were performed using the same bacterial preparations.

### Quantification of *S. aureus* with in vivo bioluminescence imaging

Mice were anesthetized via inhalation isoflurane (2%) and in vivo bioluminescence imaging was performed using an IVIS Lumina II (PerkinElmer, Hopkinton, MA).^65^ Images were obtained on POD 0, 1, 3, 5, 7, 10, 14, 18, 21, 28, 35, 42, 49, and 56. Day 56 was chosen as the endpoint for the experiment, because the unexpected delayed peak in bacterial burden around day 35 led us to prolong the experiment through to a plateau in bacterial burden, which occurred after day 56. Typical experiments previously from this lab were concluded on days 35–42, but a plateau in second peak of infection had not yet occurred by day 42. Imaging was terminated prior to day 56 in specific cases due to wound breakdown (see “Results”). Data are presented via color scale overlaid on a grayscale photograph of mice and quantified as total flux [photons per second (s) per cm^2^ (p/s/cm^2^)] within a standard circular region of interest (39 000 pixels) using Living Image software (PerkinElmer, Hopkinton, MA).

### Quantification of host immune response with in vivo fluorescence imaging

Following identical surgical procedure and inoculation in LysEGFP mice, in vivo fluorescence imaging was performed using an IVIS Lumina II (PerkinElmer, Hopkinton, MA).^65^ Images were obtained on POD 0, 1, 3, 5, 7, and 10. Data are presented via color scale overlaid on a grayscale photograph of mice and quantified as total radiant efficiency [photons per second (s) per micro-Watt per square centimeter, (p/s)/(µW/cm²)] within a standard circular region of interest tightly fit to the approximate size of implant (12 000 pixels) using Living Image software (PerkinElmer, Hopkinton, MA). The following fluorescent parameters were standardized throughout the experiment in order to control for the systemic absorption from competing sources, including hemoglobin: Exposure 2 s; Field of View 12.5; Emission Filter Open; Filter position 1; Excitation filter 465.

### Validation of the model with bacterial CFU counts

In order to confirm that the bioluminescence signal represented an accurate measure of bacterial burden, bacteria adherent to the implants and in surrounding soft tissue were quantified at the conclusion of the experiment. Bacteria were detached from the allograft by sonication in 1 mL 0.3% Tween-80 in TSB for 10 min followed by vortexing for 5 min as previously described.^31^ In addition, bacteria in the surrounding subcutaneous tissue were measured by homogenizing the encapsulating soft tissue (Pro200H Series homogenizer; Pro Scientific, Oxford, CT). The number of bacterial CFU that were adherent to the allograft and in the surrounding tissue was determined by counting CFU after overnight culture of plates, and was expressed as CFUs per mL harvested. In all cases, soft tissue was harvested that was adherent to the implant in 5 mm radius, larger than bioluminescence ROI, thereby encompassing all possible local bioluminescence burden.

### Preparation of histologic sections

In order to examine the cellular architecture of the allograft and steel implant at key time points on the bioluminescent curve, histologic sections were prepared for both sterile and 1 × 10^2^ infected groups. Wide local resection of implant and surrounding tissue was performed on POD 4, 18, 35, and 56 and high-resolution photographs were captured. Allograft specimens were fixed in 10% formalin solution overnight and then incubated in Decalcified IIH solution (Surgipath, Richmond, IL) until adequately decalcified. Stromal tissue surrounding steel discs was washed in sterile water and then fixed in 10% formalin solution overnight. Specimens were then processed and embedded in paraffin. Sagittal sections of 4 μm thickness were cut and then were stained with hematoxylin and eosin, CD31, and gram stain. CD31 is an immunohistochemistry marker of endothelial cell presence, and represents a method for identifying the microscopic vascularity of the specimen. The marker was used to assist in identifying vascular structures, which were quantified (Supplementary Fig. 11).

### Live/dead confocal microscopy

Allograft and stainless steel implants from sterile and infected mice were assessed with Live/Dead BacLight Bacterial Viability Kit (Thermo Fischer Scientific, Canoga Park, CA). This kit contains two nucleic acid stains that detect bacteria with intact plasma membrane deemed living (SYTO9—green) and bacteria with ruptured cell membrane deemed dead (Propidium iodide—red). Its use to detect living and dead bacteria has previously been reported.^66^ Specimens were analyzed and photomicrographs were recorded using the Leica DMi8 Confocal Microscope (Leica Microsystems, Buffalo Grove, IL).

### In vivo native femur transmission electron microscopy

An overnight culture of 5E6 Xen36 Staph aureus was incubated for 16 h in 10 mL TSB at 37 C. On the day of surgery, titanium pins were incubated in culture for 30 min prior to implantation. Pins were inserted in a retrograde fashion in the distal femur as described previously. On POD 14, mice were sacrificed and the femurs excised with implants intact. Femurs were immersion fixed in 0.1 mol·L^−1^ sodium cacodylate buffered 2.5% glutaraldehyde/4% paraformaldehyde overnight, followed by decalcification in 14% EDTA for 7–10 days. After decalcification, pins were removed and femurs sectioned axially. Samples were then processed for TEM beginning with postfixation in osmium tetroxide, followed by dehydration in graded acetone wash. Dehydrated samples were infiltrated with epon/acetone resin, embedded in pre-dried molds, and polymerized overnight. Embedded samples were prepared for semithin sectioning using glass knife and UCT ultramicrotome (Leica Microsystems, Buffalo Grove, IL). The thickness of the sections was 0.5 μm. Sections were mounted on glass microscope slides and stained with tolyidine blue. Area of interest was determined under light microscope and used for ultrathin sectioning with diamond knife (Diatome, Electron Microscopy Sciences, Hatfield, PA) and UCT ultramicrotome. The thickness of the sections was 55–65 nm. Sections were mounted on EM grids and stained with uranyl acetate and lead citrate. Grids were analyzed in JEOL JEM1200EX electron microscope with digital camera BioScan600W (Gatan, Pleasanton, CA). Work was performed by EM Services/EICN/CNSI.

### Statistical analysis

Each experimental group, with the exception of final pilot experiment, had at least six mice based on previous reports from our group showing that six animals/group was necessary to obtain statistical significance at the *P* < 0.05 level.^31,32^ Changes over time within and between groups were assessed using a mixed effects linear regression model of the log-transformed data with mouse random intercept. For assessment of cross-sectional differences, Student’s *t* test was used to compare the data between two groups and ANOVA was used to compare the data between three or more groups. Data are represented as mean ± standard error of the mean. Values of *P* < 0.05 were considered statistically significant. Statistical analyses were performed using Stata Statistical Software (StataCorp. 2015. Stata Statistical Software: Release 14. College Station, TX: StataCorp LP).

## Supplementary information

Supplemental Figure 1

Supplemental Figure 2

Supplemental Figure 3

Supplemental Figure 4

Supplemental Figure 5

Supplemental Figure 6

Supplemental Figure 7

Supplemental Figure 8

Supplemental Figure 9

Supplemental Figure 10

Supplemental Figure 11

Supplemental Figure 12

## Data Availability

All data represented herein will be promptly made available to readers upon request without undue qualifications.
