# Supplementary material for: Evading the host response: Staphylococcus “hiding” in cortical bone canalicular system causes increased bacterial burden
Source: Bone Res. 2020 Dec 10;8:43. doi: 10.1038/s41413-020-00118-w (PMC7728749; doi:10.1038/s41413-020-00118-w)
Supplement: Supplementary file 1 — Supplemental Figure 1 [file 41413_2020_118_MOESM1_ESM.pptx]

## Slide 1
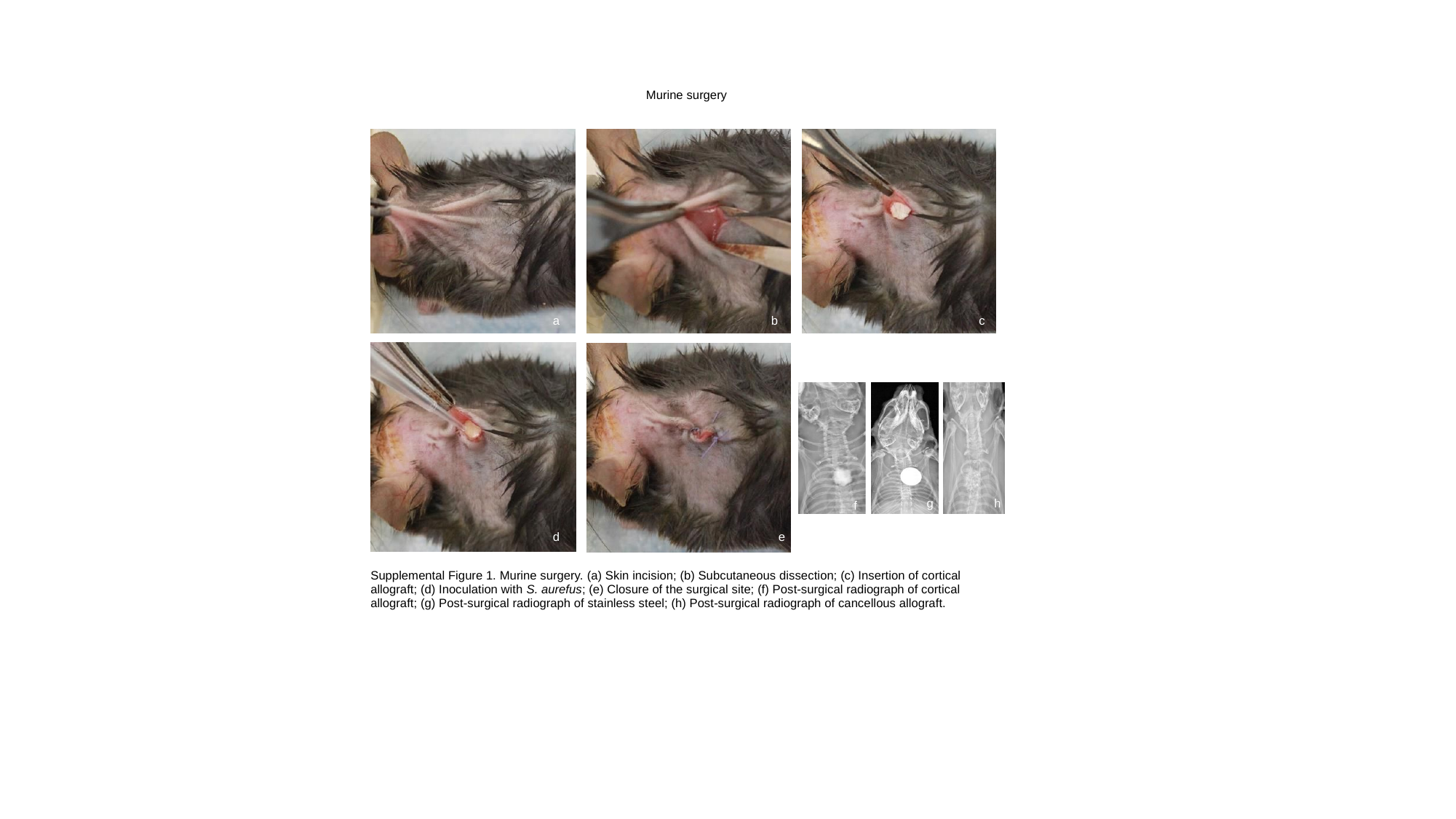

| Murine surgery |
| --- |
| Supplemental Figure 1. Murine surgery. (a) Skin incision; (b) Subcutaneous dissection; (c) Insertion of cortical allograft; (d) Inoculation with S. aurefus; (e) Closure of the surgical site; (f) Post-surgical radiograph of cortical allograft; (g) Post-surgical radiograph of stainless steel; (h) Post-surgical radiograph of cancellous allograft. |
a
b
c
g
h
f
d
e
