# Supplementary material for: Evading the host response: Staphylococcus “hiding” in cortical bone canalicular system causes increased bacterial burden
Source: Bone Res. 2020 Dec 10;8:43. doi: 10.1038/s41413-020-00118-w (PMC7728749; doi:10.1038/s41413-020-00118-w)
Supplement: Supplementary file 2 — Supplemental Figure 2 [file 41413_2020_118_MOESM2_ESM.pptx]

## Slide 1
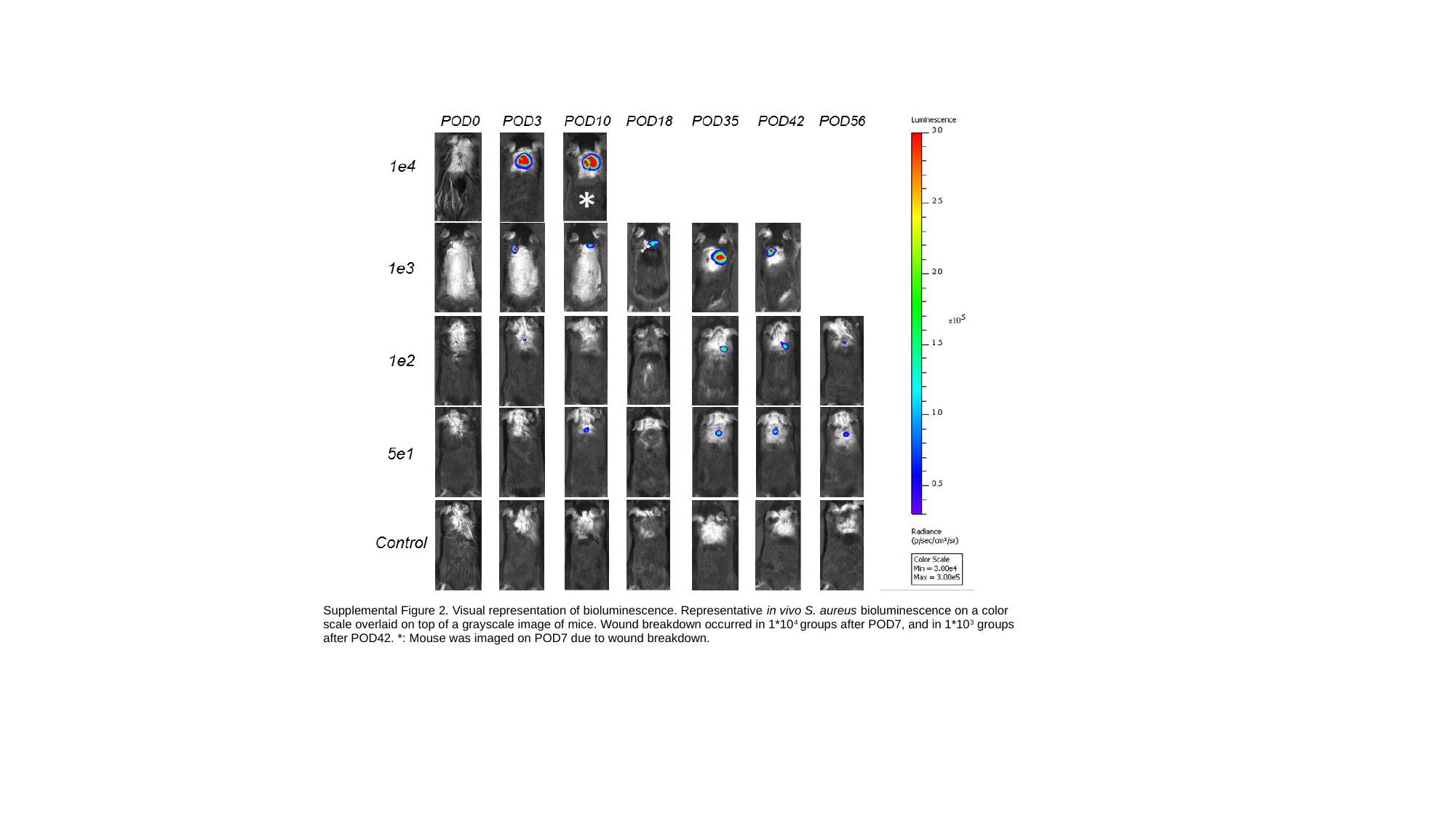

| |
| --- |
| Supplemental Figure 2. Visual representation of bioluminescence. Representative in vivo S. aureus bioluminescence on a color scale overlaid on top of a grayscale image of mice. Wound breakdown occurred in 1\*104 groups after POD7, and in 1\*103 groups after POD42. \*: Mouse was imaged on POD7 due to wound breakdown. |
