# Supplementary material for: Evading the host response: Staphylococcus “hiding” in cortical bone canalicular system causes increased bacterial burden
Source: Bone Res. 2020 Dec 10;8:43. doi: 10.1038/s41413-020-00118-w (PMC7728749; doi:10.1038/s41413-020-00118-w)
Supplement: Supplementary file 4 — Supplemental Figure 4 [file 41413_2020_118_MOESM4_ESM.pptx]

## Slide 1
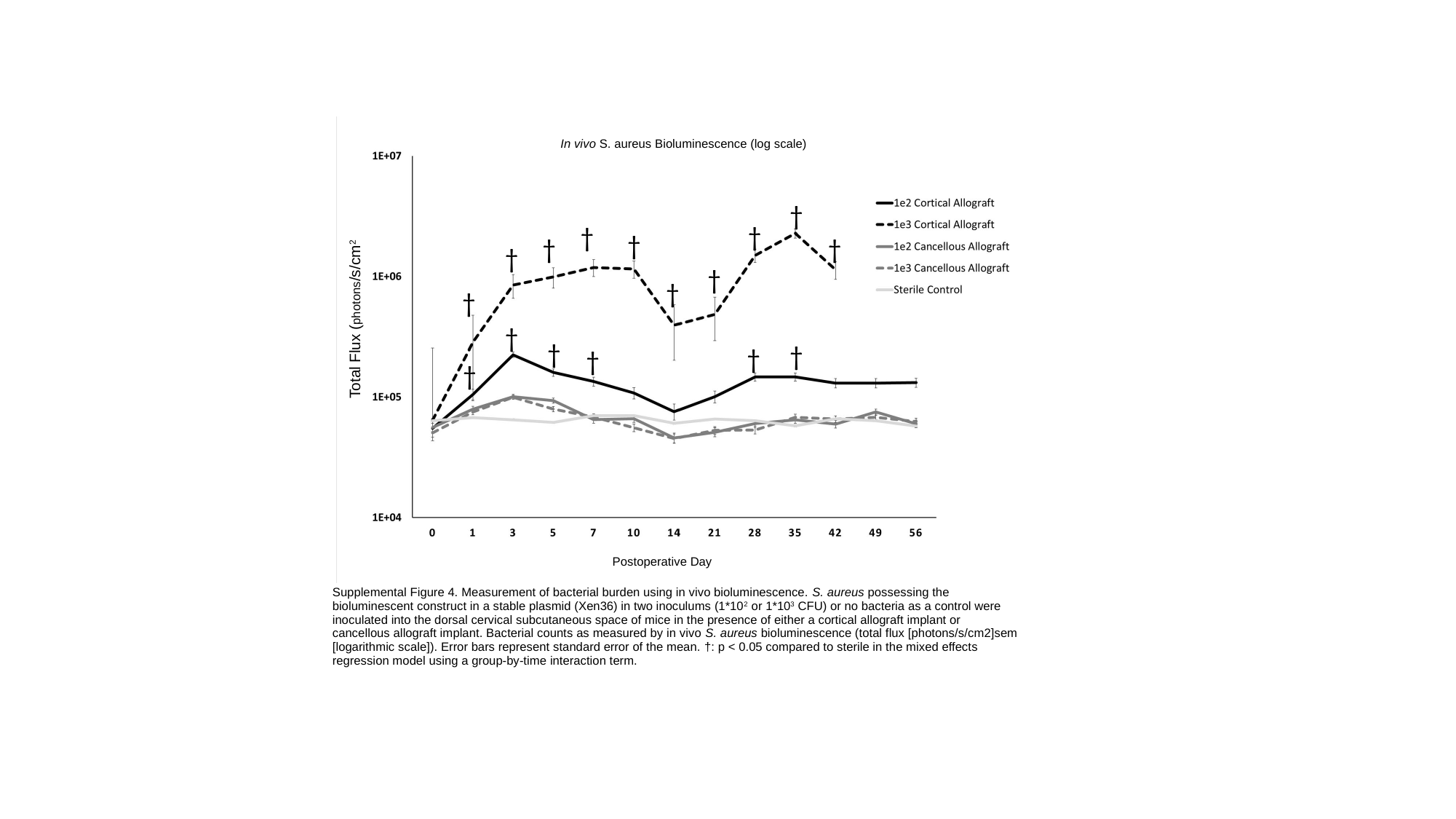

| |
| --- |
| Supplemental Figure 4. Measurement of bacterial burden using in vivo bioluminescence. S. aureus possessing the bioluminescent construct in a stable plasmid (Xen36) in two inoculums (1\*102 or 1\*103 CFU) or no bacteria as a control were inoculated into the dorsal cervical subcutaneous space of mice in the presence of either a cortical allograft implant or cancellous allograft implant. Bacterial counts as measured by in vivo S. aureus bioluminescence (total flux [photons/s/cm2]sem [logarithmic scale]). Error bars represent standard error of the mean. †: p < 0.05 compared to sterile in the mixed effects regression model using a group-by-time interaction term. |
 In vivo S. aureus Bioluminescence (log scale)
†
†
†
†
†
†
†
†
†
†
 Total Flux (photons/s/cm2
†
†
†
†
†
†
 Postoperative Day
