# Supplementary material for: Evading the host response: Staphylococcus “hiding” in cortical bone canalicular system causes increased bacterial burden
Source: Bone Res. 2020 Dec 10;8:43. doi: 10.1038/s41413-020-00118-w (PMC7728749; doi:10.1038/s41413-020-00118-w)
Supplement: Supplementary file 5 — Supplemental Figure 5 [file 41413_2020_118_MOESM5_ESM.pptx]

## Slide 1
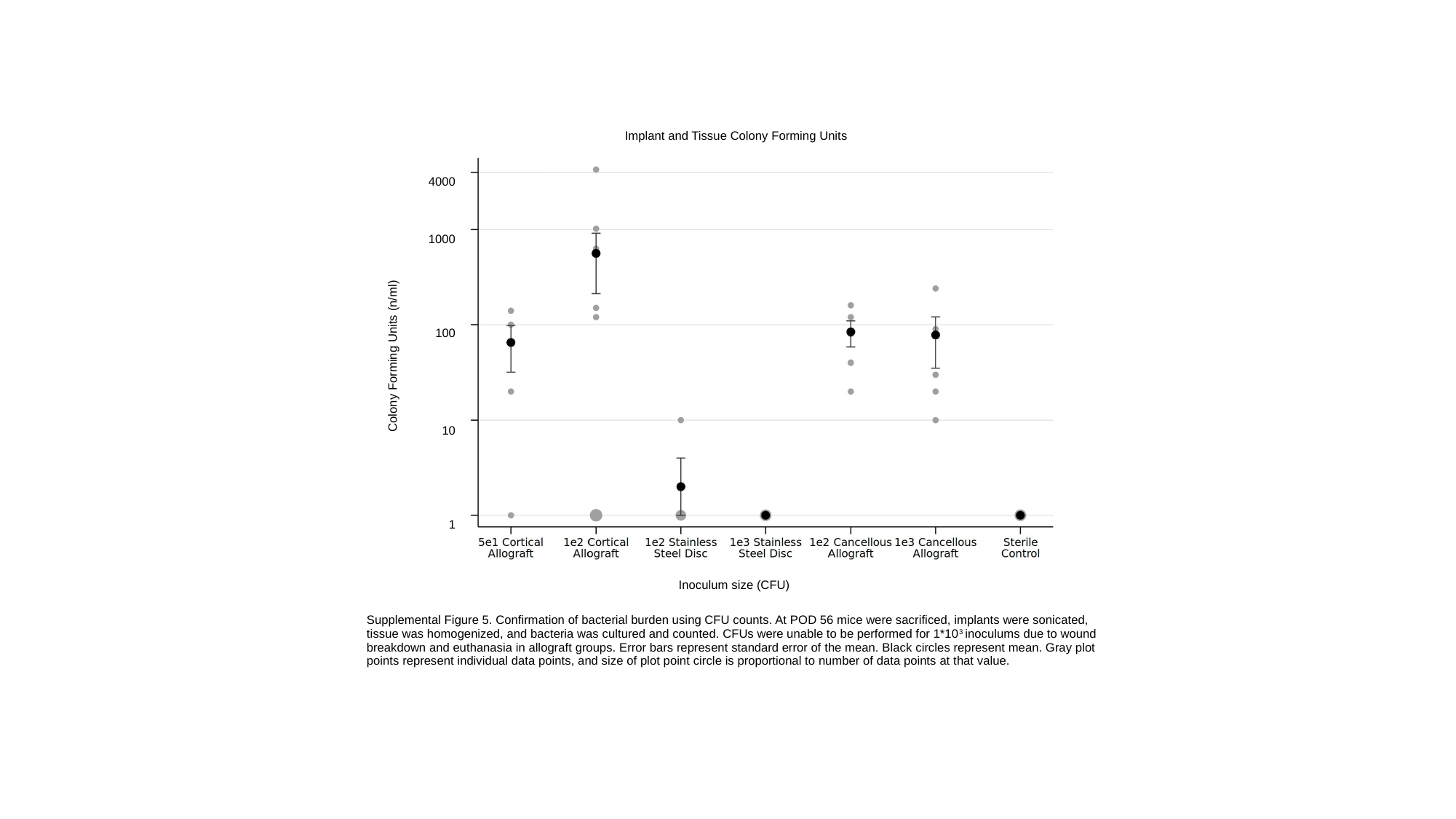

| |
| --- |
| Supplemental Figure 5. Confirmation of bacterial burden using CFU counts. At POD 56 mice were sacrificed, implants were sonicated, tissue was homogenized, and bacteria was cultured and counted. CFUs were unable to be performed for 1\*103 inoculums due to wound breakdown and euthanasia in allograft groups. Error bars represent standard error of the mean. Black circles represent mean. Gray plot points represent individual data points, and size of plot point circle is proportional to number of data points at that value. |
Implant and Tissue Colony Forming Units
4000
1000
100
10
1
Colony Forming Units (n/ml)
Inoculum size (CFU)
