# Supplementary material for: Evading the host response: Staphylococcus “hiding” in cortical bone canalicular system causes increased bacterial burden
Source: Bone Res. 2020 Dec 10;8:43. doi: 10.1038/s41413-020-00118-w (PMC7728749; doi:10.1038/s41413-020-00118-w)
Supplement: Supplementary file 6 — Supplemental Figure 6 [file 41413_2020_118_MOESM6_ESM.pptx]

## Slide 1
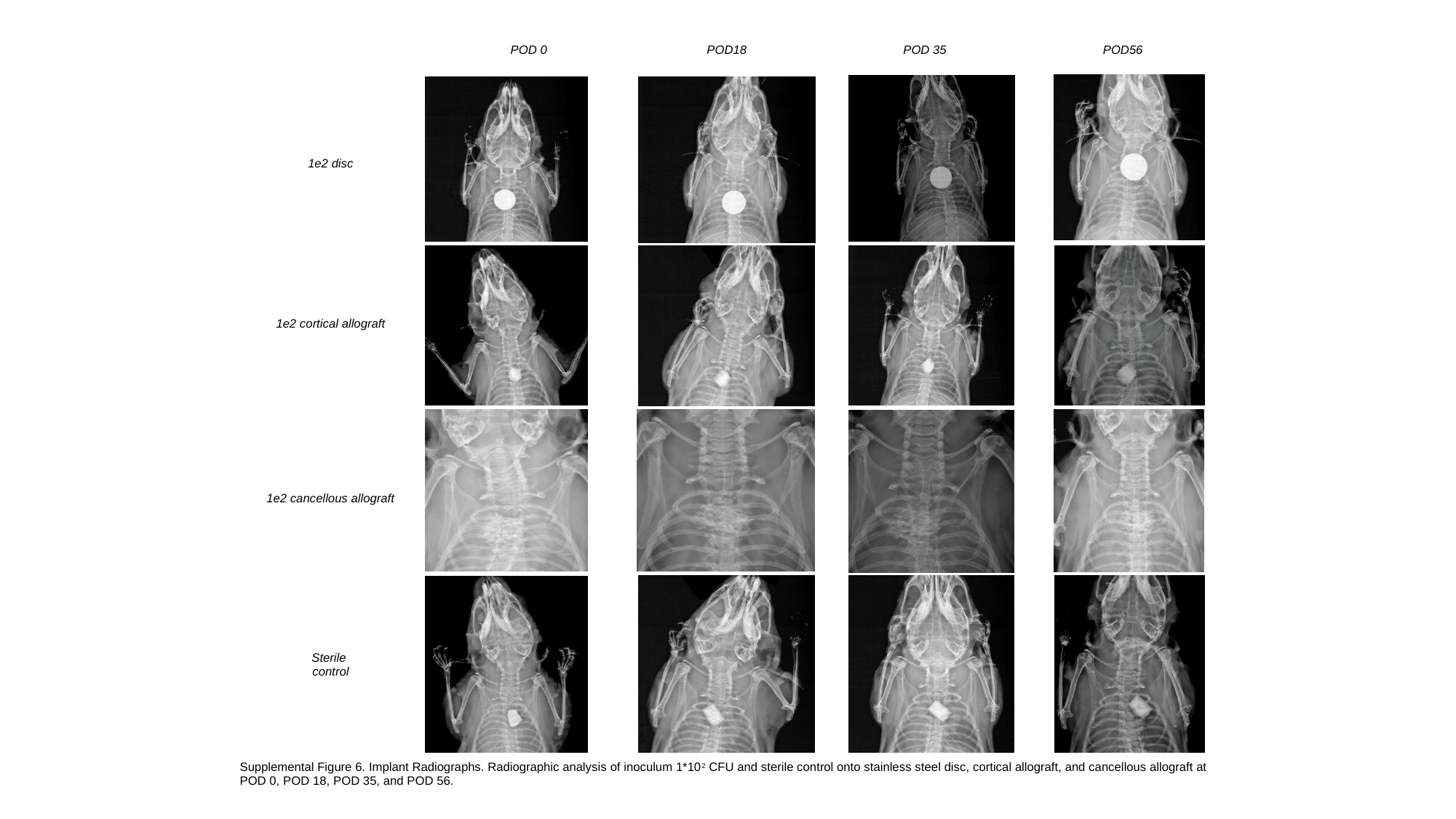

| | POD 0 | POD18 | POD 35 | POD56 |
| --- | --- | --- | --- | --- |
| 1e2 disc | | | | |
| 1e2 cortical allograft | | | | |
| 1e2 cancellous allograft | | | | |
| Sterile control | | | | |
| Supplemental Figure 6. Implant Radiographs. Radiographic analysis of inoculum 1\*102 CFU and sterile control onto stainless steel disc, cortical allograft, and cancellous allograft at POD 0, POD 18, POD 35, and POD 56. | | | | |
