# Supplementary material for: Evading the host response: Staphylococcus “hiding” in cortical bone canalicular system causes increased bacterial burden
Source: Bone Res. 2020 Dec 10;8:43. doi: 10.1038/s41413-020-00118-w (PMC7728749; doi:10.1038/s41413-020-00118-w)
Supplement: Supplementary file 7 — Supplemental Figure 7 [file 41413_2020_118_MOESM7_ESM.pptx]

## Slide 1
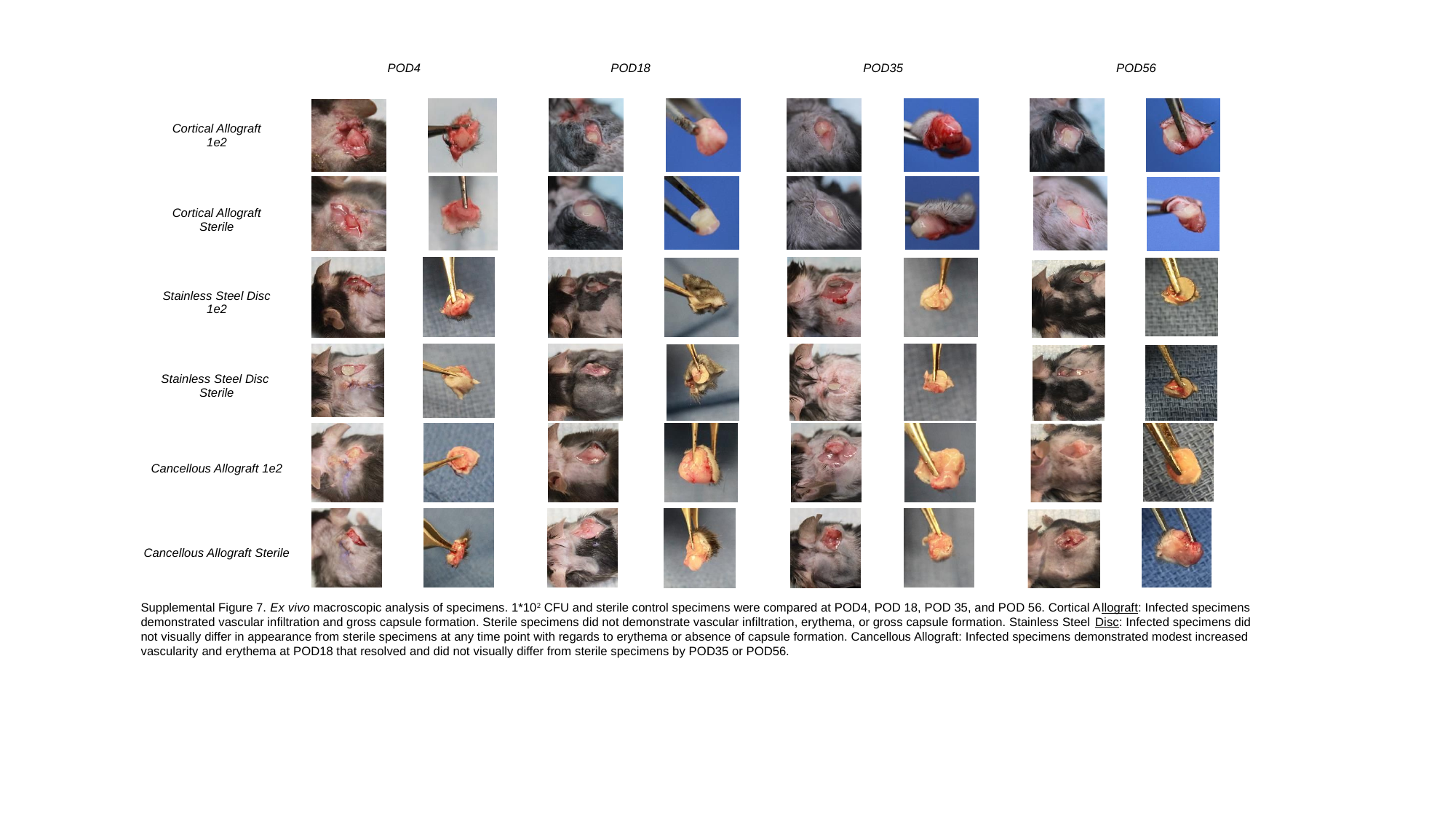

| | POD4 | POD18 | POD35 | POD56 |
| --- | --- | --- | --- | --- |
| Cortical Allograft 1e2 | | | | |
| Cortical Allograft Sterile | | | | |
| Stainless Steel Disc 1e2 | | | | |
| Stainless Steel Disc Sterile | | | | |
| Cancellous Allograft 1e2 | | | | |
| Cancellous Allograft Sterile | | | | |
Supplemental Figure 7. Ex vivo macroscopic analysis of specimens. 1*102 CFU and sterile control specimens were compared at POD4, POD 18, POD 35, and POD 56. Cortical Allograft: Infected specimens demonstrated vascular infiltration and gross capsule formation. Sterile specimens did not demonstrate vascular infiltration, erythema, or gross capsule formation. Stainless Steel Disc: Infected specimens did not visually differ in appearance from sterile specimens at any time point with regards to erythema or absence of capsule formation. Cancellous Allograft: Infected specimens demonstrated modest increased vascularity and erythema at POD18 that resolved and did not visually differ from sterile specimens by POD35 or POD56.
