# Supplementary material for: Evading the host response: Staphylococcus “hiding” in cortical bone canalicular system causes increased bacterial burden
Source: Bone Res. 2020 Dec 10;8:43. doi: 10.1038/s41413-020-00118-w (PMC7728749; doi:10.1038/s41413-020-00118-w)
Supplement: Supplementary file 8 — Supplemental Figure 8 [file 41413_2020_118_MOESM8_ESM.pptx]

## Slide 1
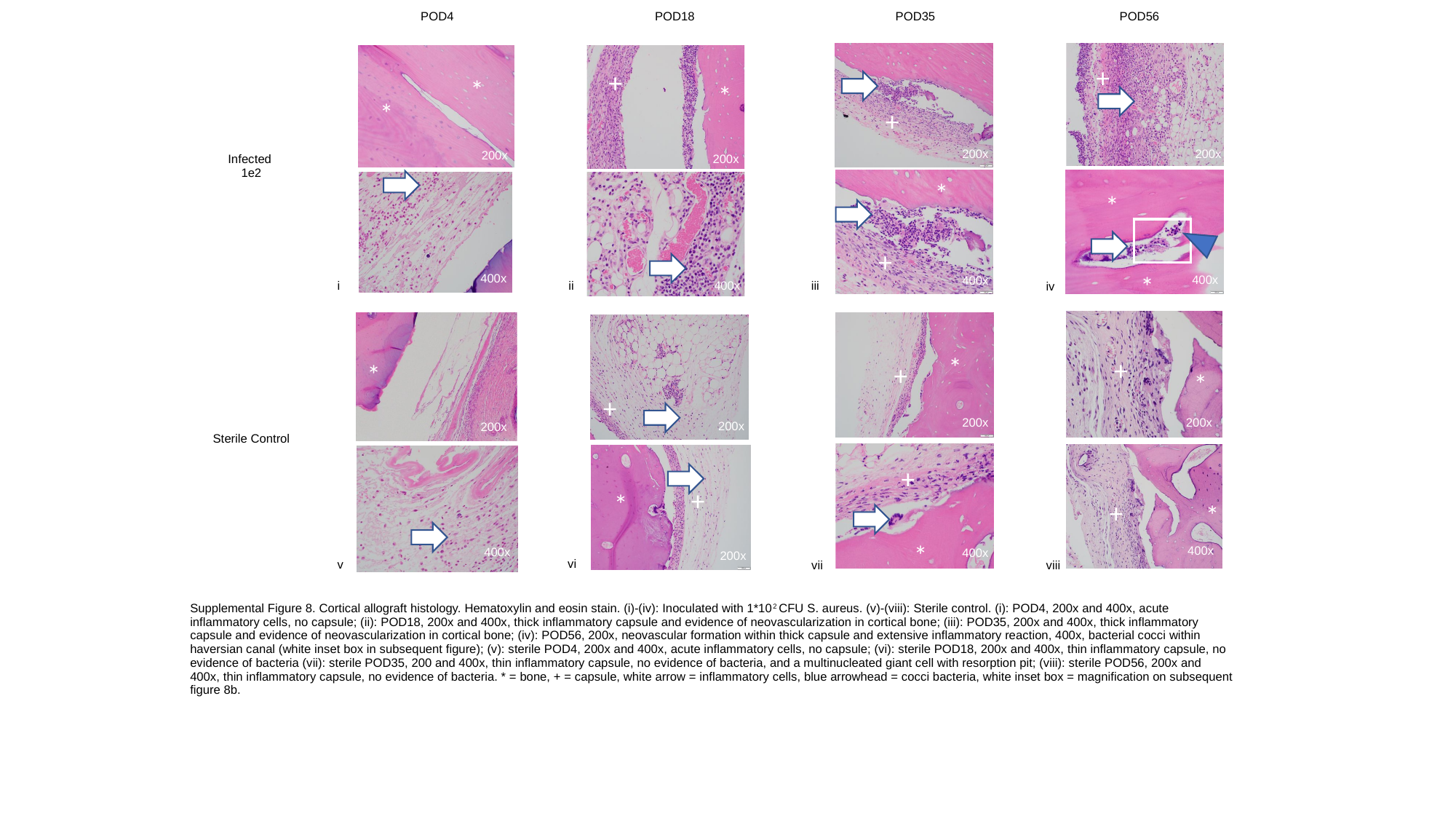

| | POD4 | POD18 | POD35 | POD56 |
| --- | --- | --- | --- | --- |
| Infected 1e2 | | | | |
| Sterile Control | | | | |
| Supplemental Figure 8. Cortical allograft histology. Hematoxylin and eosin stain. (i)-(iv): Inoculated with 1\*102 CFU S. aureus. (v)-(viii): Sterile control. (i): POD4, 200x and 400x, acute inflammatory cells, no capsule; (ii): POD18, 200x and 400x, thick inflammatory capsule and evidence of neovascularization in cortical bone; (iii): POD35, 200x and 400x, thick inflammatory capsule and evidence of neovascularization in cortical bone; (iv): POD56, 200x, neovascular formation within thick capsule and extensive inflammatory reaction, 400x, bacterial cocci within haversian canal (white inset box in subsequent figure); (v): sterile POD4, 200x and 400x, acute inflammatory cells, no capsule; (vi): sterile POD18, 200x and 400x, thin inflammatory capsule, no evidence of bacteria (vii): sterile POD35, 200 and 400x, thin inflammatory capsule, no evidence of bacteria, and a multinucleated giant cell with resorption pit; (viii): sterile POD56, 200x and 400x, thin inflammatory capsule, no evidence of bacteria. \* = bone, + = capsule, white arrow = inflammatory cells, blue arrowhead = cocci bacteria, white inset box = magnification on subsequent figure 8b. | | | | |
+
+
*
*
*
+
200x
200x
200x
200x
*
*
+
*
400x
 400x
400x
i
ii
iii
400x
iv
*
+
*
+
*
+
200x
200x
200x
200x
+
+
*
+
*
*
400x
400x
400x
200x
vi
v
vii
viii
