# Supplementary material for: Evading the host response: Staphylococcus “hiding” in cortical bone canalicular system causes increased bacterial burden
Source: Bone Res. 2020 Dec 10;8:43. doi: 10.1038/s41413-020-00118-w (PMC7728749; doi:10.1038/s41413-020-00118-w)
Supplement: Supplementary file 9 — Supplemental Figure 9 [file 41413_2020_118_MOESM9_ESM.pptx]

## Slide 1
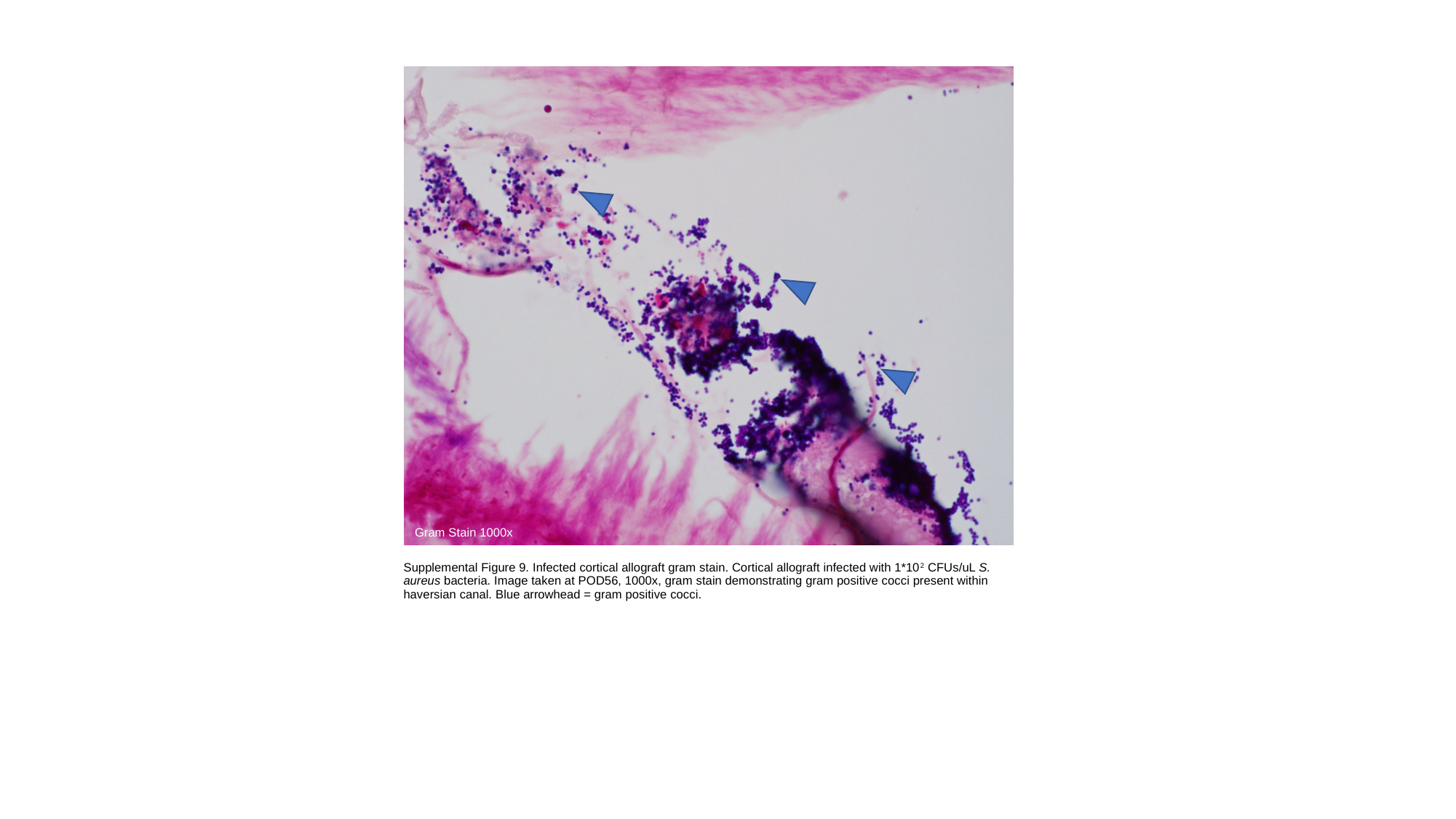

| |
| --- |
| Supplemental Figure 9. Infected cortical allograft gram stain. Cortical allograft infected with 1\*102 CFUs/uL S. aureus bacteria. Image taken at POD56, 1000x, gram stain demonstrating gram positive cocci present within haversian canal. Blue arrowhead = gram positive cocci. |
Gram Stain 1000x
