# Supplementary material for: Evading the host response: Staphylococcus “hiding” in cortical bone canalicular system causes increased bacterial burden
Source: Bone Res. 2020 Dec 10;8:43. doi: 10.1038/s41413-020-00118-w (PMC7728749; doi:10.1038/s41413-020-00118-w)
Supplement: Supplementary file 10 — Supplemental Figure 10 [file 41413_2020_118_MOESM10_ESM.pptx]

## Slide 1
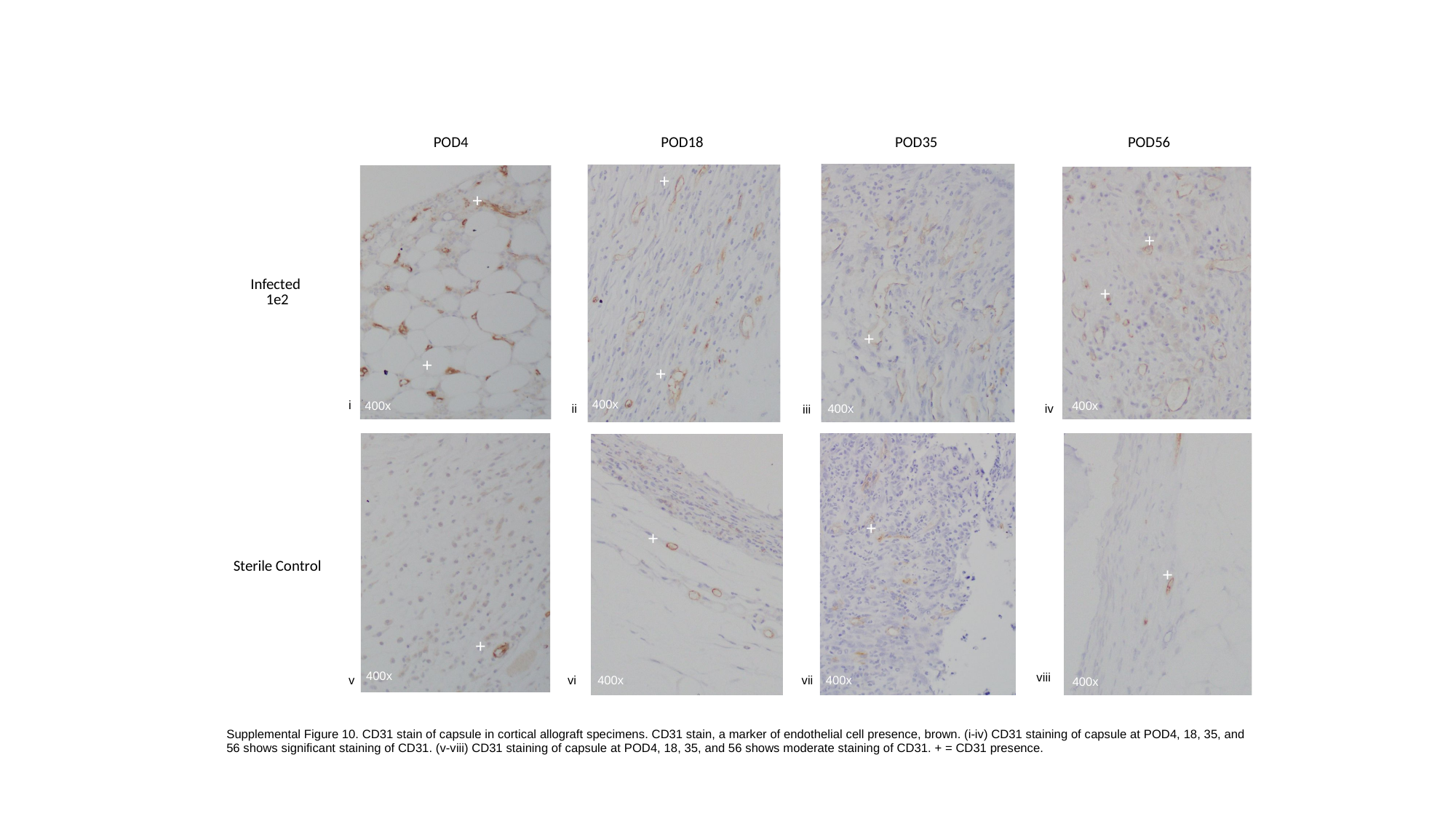

| | POD4 | POD18 | POD35 | POD56 |
| --- | --- | --- | --- | --- |
| Infected 1e2 | | | | |
| Sterile Control | | | | |
| Supplemental Figure 10. CD31 stain of capsule in cortical allograft specimens. CD31 stain, a marker of endothelial cell presence, brown. (i-iv) CD31 staining of capsule at POD4, 18, 35, and 56 shows significant staining of CD31. (v-viii) CD31 staining of capsule at POD4, 18, 35, and 56 shows moderate staining of CD31. + = CD31 presence. | | | | |
+
+
+
+
+
200x
+
+
400x
i
400x
400x
ii
400x
iv
iii
+
+
+
+
400x
viii
v
vi
vii
400x
400x
400x
