# Supplementary material for: Evading the host response: Staphylococcus “hiding” in cortical bone canalicular system causes increased bacterial burden
Source: Bone Res. 2020 Dec 10;8:43. doi: 10.1038/s41413-020-00118-w (PMC7728749; doi:10.1038/s41413-020-00118-w)
Supplement: Supplementary file 11 — Supplemental Figure 11 [file 41413_2020_118_MOESM11_ESM.pptx]

## Slide 1
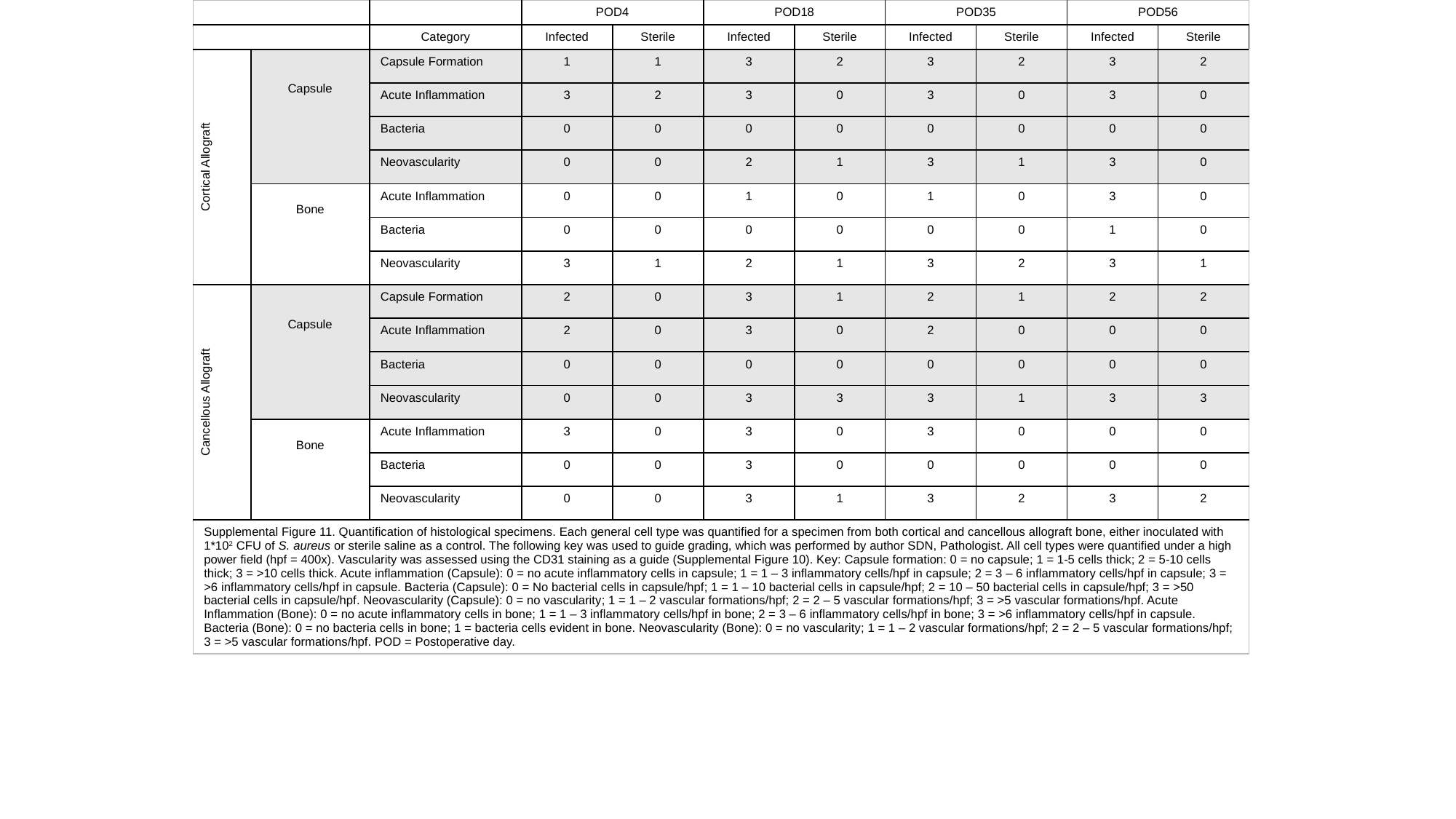

| | | | POD4 | | POD18 | | POD35 | | POD56 | |
| --- | --- | --- | --- | --- | --- | --- | --- | --- | --- | --- |
| | | Category | Infected | Sterile | Infected | Sterile | Infected | Sterile | Infected | Sterile |
| Cortical Allograft | Capsule | Capsule Formation | 1 | 1 | 3 | 2 | 3 | 2 | 3 | 2 |
| | | Acute Inflammation | 3 | 2 | 3 | 0 | 3 | 0 | 3 | 0 |
| | | Bacteria | 0 | 0 | 0 | 0 | 0 | 0 | 0 | 0 |
| | | Neovascularity | 0 | 0 | 2 | 1 | 3 | 1 | 3 | 0 |
| | Bone | Acute Inflammation | 0 | 0 | 1 | 0 | 1 | 0 | 3 | 0 |
| | | Bacteria | 0 | 0 | 0 | 0 | 0 | 0 | 1 | 0 |
| | | Neovascularity | 3 | 1 | 2 | 1 | 3 | 2 | 3 | 1 |
| Cancellous Allograft | Capsule | Capsule Formation | 2 | 0 | 3 | 1 | 2 | 1 | 2 | 2 |
| | | Acute Inflammation | 2 | 0 | 3 | 0 | 2 | 0 | 0 | 0 |
| | | Bacteria | 0 | 0 | 0 | 0 | 0 | 0 | 0 | 0 |
| | | Neovascularity | 0 | 0 | 3 | 3 | 3 | 1 | 3 | 3 |
| | Bone | Acute Inflammation | 3 | 0 | 3 | 0 | 3 | 0 | 0 | 0 |
| | | Bacteria | 0 | 0 | 3 | 0 | 0 | 0 | 0 | 0 |
| | | Neovascularity | 0 | 0 | 3 | 1 | 3 | 2 | 3 | 2 |
| Supplemental Figure 11. Quantification of histological specimens. Each general cell type was quantified for a specimen from both cortical and cancellous allograft bone, either inoculated with 1\*102 CFU of S. aureus or sterile saline as a control. The following key was used to guide grading, which was performed by author SDN, Pathologist. All cell types were quantified under a high power field (hpf = 400x). Vascularity was assessed using the CD31 staining as a guide (Supplemental Figure 10). Key: Capsule formation: 0 = no capsule; 1 = 1-5 cells thick; 2 = 5-10 cells thick; 3 = >10 cells thick. Acute inflammation (Capsule): 0 = no acute inflammatory cells in capsule; 1 = 1 – 3 inflammatory cells/hpf in capsule; 2 = 3 – 6 inflammatory cells/hpf in capsule; 3 = >6 inflammatory cells/hpf in capsule. Bacteria (Capsule): 0 = No bacterial cells in capsule/hpf; 1 = 1 – 10 bacterial cells in capsule/hpf; 2 = 10 – 50 bacterial cells in capsule/hpf; 3 = >50 bacterial cells in capsule/hpf. Neovascularity (Capsule): 0 = no vascularity; 1 = 1 – 2 vascular formations/hpf; 2 = 2 – 5 vascular formations/hpf; 3 = >5 vascular formations/hpf. Acute Inflammation (Bone): 0 = no acute inflammatory cells in bone; 1 = 1 – 3 inflammatory cells/hpf in bone; 2 = 3 – 6 inflammatory cells/hpf in bone; 3 = >6 inflammatory cells/hpf in capsule. Bacteria (Bone): 0 = no bacteria cells in bone; 1 = bacteria cells evident in bone. Neovascularity (Bone): 0 = no vascularity; 1 = 1 – 2 vascular formations/hpf; 2 = 2 – 5 vascular formations/hpf; 3 = >5 vascular formations/hpf. POD = Postoperative day. | | | | | | | | | | |
