# Supplementary material for: Evading the host response: Staphylococcus “hiding” in cortical bone canalicular system causes increased bacterial burden
Source: Bone Res. 2020 Dec 10;8:43. doi: 10.1038/s41413-020-00118-w (PMC7728749; doi:10.1038/s41413-020-00118-w)
Supplement: Supplementary file 12 — Supplemental Figure 12 [file 41413_2020_118_MOESM12_ESM.pptx]

## Slide 1
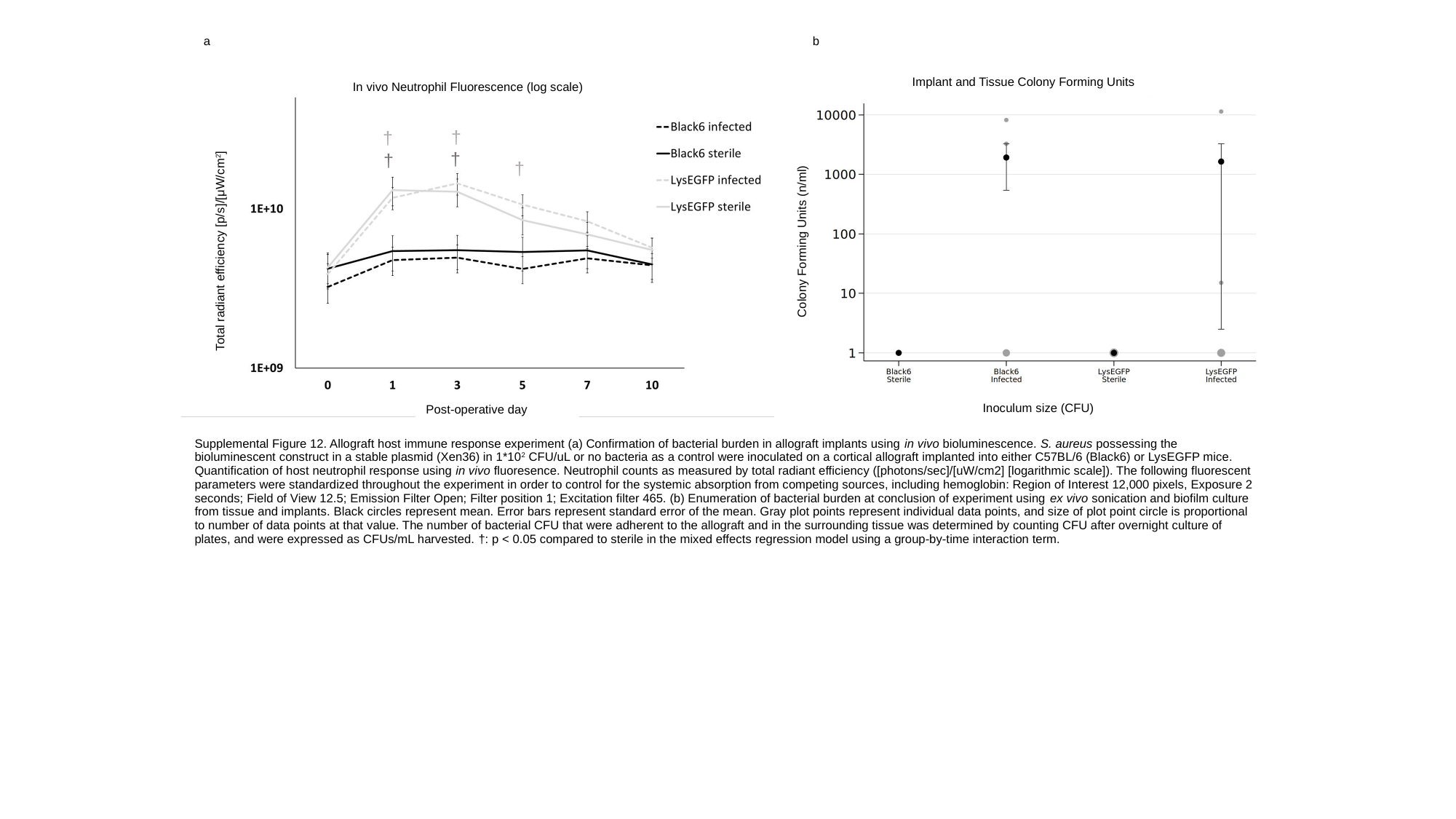

| |
| --- |
| Supplemental Figure 12. Allograft host immune response experiment (a) Confirmation of bacterial burden in allograft implants using in vivo bioluminescence. S. aureus possessing the bioluminescent construct in a stable plasmid (Xen36) in 1\*102 CFU/uL or no bacteria as a control were inoculated on a cortical allograft implanted into either C57BL/6 (Black6) or LysEGFP mice. Quantification of host neutrophil response using in vivo fluoresence. Neutrophil counts as measured by total radiant efficiency ([photons/sec]/[uW/cm2] [logarithmic scale]). The following fluorescent parameters were standardized throughout the experiment in order to control for the systemic absorption from competing sources, including hemoglobin: Region of Interest 12,000 pixels, Exposure 2 seconds; Field of View 12.5; Emission Filter Open; Filter position 1; Excitation filter 465. (b) Enumeration of bacterial burden at conclusion of experiment using ex vivo sonication and biofilm culture from tissue and implants. Black circles represent mean. Error bars represent standard error of the mean. Gray plot points represent individual data points, and size of plot point circle is proportional to number of data points at that value. The number of bacterial CFU that were adherent to the allograft and in the surrounding tissue was determined by counting CFU after overnight culture of plates, and were expressed as CFUs/mL harvested. †: p < 0.05 compared to sterile in the mixed effects regression model using a group-by-time interaction term. |
a
b
Implant and Tissue Colony Forming Units
 In vivo Neutrophil Fluorescence (log scale)
†
†
†
†
†
Colony Forming Units (n/ml)
 Total radiant efficiency [p/s]/[μW/cm2]
Inoculum size (CFU)
Post-operative day
